# New Rare *Ent*-Clerodane Diterpene Peroxides from Egyptian Mountain Tea (Qourtom) and Its Chemosystem as Herbal Remedies and Phytonutrients Agents

**DOI:** 10.3390/molecules25092172

**Published:** 2020-05-06

**Authors:** Taha A. Hussien, Ahmed A. Mahmoud, Naglaa S. Mohamed, Abdelaaty A. Shahat, Hesham R. El-Seedi, Mohamed-Elamir F. Hegazy

**Affiliations:** 1 Pharmacognosy Department, Faculty of Pharmacy, Deraya University, El-Minia 61519, Egypt; thussien71@yahoo.com; 2 Chemistry Department, Faculty of Science, Minia University, El-Minia 61519, Egypt; 3 Chemistry Department, Faculty of Science, Aswan University, Aswan 81528, Egypt; naglaanaglaa@yahoo.com; 4 Pharmacognosy Department, College of Pharmacy, King Saud University, P.O. Box 2457, Riyadh 11451, Saudi Arabia; ashahat@ksu.edu.sa; 5 Chemistry of Medicinal Plants Department, National Research Centre, 33 El-Bohouth St., Dokki, Giza 12622, Egypt; 6 Department of Molecular Biosciences, The Wenner-Gren Institute, Stockholm University, S-106 91 Stockholm, Sweden; 7 International Research Center for Food Nutrition and Safety, Jiangsu University, Zhenjiang 212013, China; 8 Al-Rayan Research and Innovation Center, Al-Rayan Colleges, Medina 42541, Saudi Arabia; 9 Pharmacognosy Group, Department of Medicinal Chemistry, Uppsala University, Biomedical Centre, Box 574, 75123 Uppsala, Sweden

**Keywords:** *Stachys aegyptiaca*, lamiaceae, herbal tea, nutrients, *neo*-clerodane diterpene peroxides

## Abstract

Genus *Stachys*, the largest genera of the family Lamiaceae, and its species are frequently used as herbal teas due to their essential oils. Tubers of some *Stachys* species are also consumed as important nutrients for humans and animals due to their carbohydrate contents. Three new *neo*-clerodane diterpene peroxides, named stachaegyptin F-H (**1, 2,** and **4**), together with two known compounds, stachysperoxide (**3**) and stachaegyptin A (**5**), were isolated from *Stachys aegyptiaca* aerial parts. Their structures were determined using a combination of spectroscopic techniques, including HR-FAB-MS and extensive 1D and 2D NMR (^1^H, ^13^C NMR, DEPT, ^1^H-^1^H COSY, HMQC, HMBC and NOESY) analyses. Additionally, a biosynthetic pathway for the isolated compounds (**1**–**5**) was discussed. The chemotaxonomic significance of the isolated diterpenoids of *S. aegyptiaca* in comparison to the previous reported ones from other *Stachys* species was also studied.

## 1. Introduction

The genus *Stachys* (woundwort) has about 300 species growing wild in the temperate and tropical regions throughout the world except the continent of Australia and New Zealand [1]. In the Mediterranean region and Iran, *Stachys* species are known as mountain tea with great medicinal and nutritional values due to their traditional uses as food additives, herbal teas, and medicinal supplements [2,3,4,5]. The tubers of some species are used as phytonutrients rich in carbohydrates, particularly in some parts of Europe and China [6]. In folk medicine, the infusions, decoctions, and ointments made from flowers and leaves of these herbs have been used in the treatment of some disorders such as skin infections, inflammation, wounds, digestive problems, cough, ulcers, and stomach ache, and applied as antispasmodic, sedative, and diuretic agents, and cardiac tonic [3,5,7,8,9,10], and recently administrated for genital tumours, sclerosis of the spleen, and inflammatory cancerous ulcers [11,12,13]. Phenolic extracts and essential oils of *Stachys* species showed a number of important biological activities such as antioxidant [14,15,16,17,18], anti-inflammatory [16,19], antiangiogenic [20], anti-nociceptive [21,22], antimicrobial [3,4,23,24], cytotoxic, and anticancer [25,26,27,28,29,30]. Additionally, the genus *Stachys* is rich with flavonoids and phenolic [17,31,32,33,34,35,36], diterpenoids [10,21,27,37,38,39,40,41,42], iridoids [20,43,44,45], and phenylethanoid glycosides [46,47] metabolites.

*Stachys aegyptiaca* Pers., a member of this genus, is a perennial aromatic plant growing wild in Sinai Peninsula, Egypt, and is called “Qourtom”. Previous phytochemical investigations on this species led to the isolation of diterpenes [27,40,41,48], flavonoids [40,49,50,51,52]), and essential oils [53,54]. In our previous work on this species, we isolated five new diterpenes of the *neo*-clerodane type, stachaegyptin A-E, in addition to seven known flavonoids from the aerial parts [27,40].

**Figure 1:**
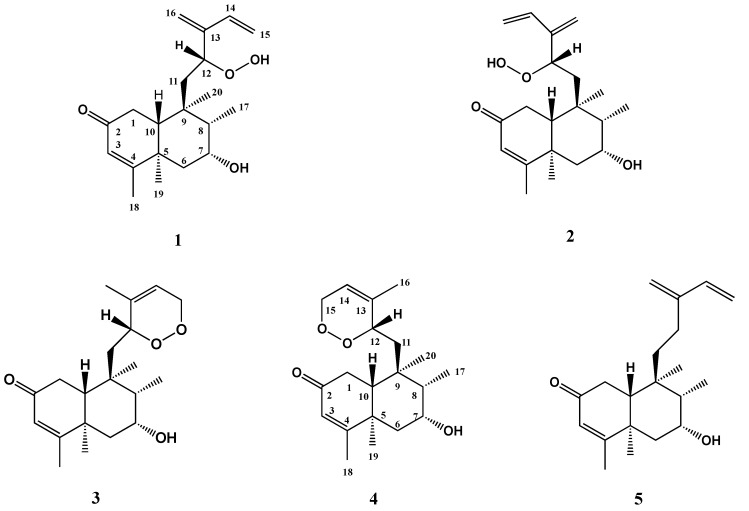
Structures of the isolated diterpenes from *Stachys aegyptiaca.*

Herein, we report the isolation and structural determination of further three new *ent*-*neo*-clerodane diterpene peroxides, named stachaegyptin F-H (**1, 2, 4**), as well as two known compounds, stachysperoxide (**3**) and stachaegyptin A (**5**) (Figure 1), from the aerial parts of this species using extensive 1D and 2D NMR and HR-FAB-MS analyses. Additionally, a biosynthetic pathway of the isolated metabolites (**1**–**5**) as well as the chemotaxonomic significance of the isolated diterpenoids from *S. aegyptiaca* were studied.

## 2. Results and Discussion

The CH_2_Cl_2_:MeOH (1:1) extract of *S. aegyptiaca* aerial parts afforded three new *ent*-*neo*-clerodane diterpenoids, named stachaegyptin F (**1**), stachaegyptin G (**2**), and stachaegyptin H (**4**), together with two known compounds, stachysperoxide (**3**) and stachaegyptin A (**5**) (Figure 1), using chromatographic techniques. Their structures were established using extensive 1D [^1^H (Table 1), ^13^C NMR (Table 2)], and 2D NMR (^1^H-^1^H COSY, HMQC, HMBC and NOESY) analyses(the details in Supplementary Materials).

**Figure 2:**
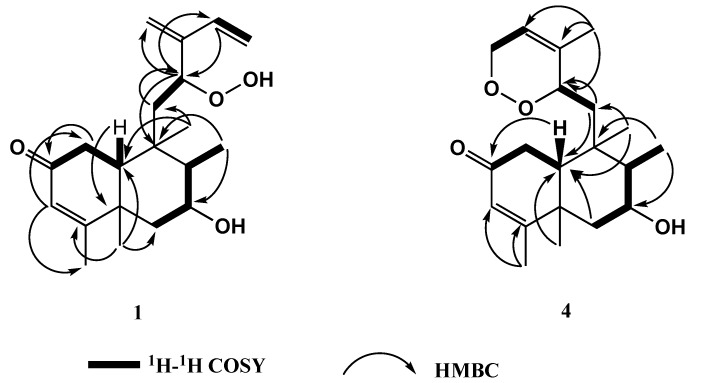
Observed ^1^H-^1^H-COSY and HMBC correlations for **1** and **4.**

Compound **1** was isolated as a colorless oil with an optical rotation of ${\left[\alpha \right]}_{D}^{25}$+30 (*c*, 0.001, MeOH). Its molecular formula C_20_H_30_O_4_ was determined from the high-resolution FAB-MS analysis with a molecular ion peak [M + Na]^+^ at *m*/*z* 357.2045 (calcd. for C_20_H_30_O_4_Na, 357.2044), indicating six degrees of unsaturation. The ^13^C NMR spectrum revealed the presence of 20 carbon resonances (Table 2), which was in agreement with the molecular formula. Their multiplicities were deduced from the results of ^13^C DEPT NMR analyses as four methyls, five methylenes (two olefinic), six methines (two olefinic and two oxygenated at δ_C_ 73.2 and δ_C_ 83.7), and five quaternary carbons (two olefinic and one keto at δ_C_ 199.7) (Table 2). With 20 carbons and six degrees of unsaturation; one of them was assigned as a keto group (δ_C_ 199.8) and three were attributed to double bonds, therefore, compound **1** is apparently a bicyclic diterpene. The ^1^H NMR analysis of **1** (Table 1) displayed typical signals for two tertiary methyls at δ_H_ 1.02 and 1.39 (each 3H, *s*), a secondary methyl at δ_H_ 1.09 (3H, *d*, *J* = 7.0 Hz) and an olefinic methyl at δ_H_ 1.92 (3H, *s*), which showed a correlation in the Double Quantum Filtered COSY (DQF-COSY) spectrum with an olefinic proton signal at δ_H_ 5.68 (1H, *br s*), indicating the presence of a trisubstituted double bond. The spectrum also showed two oxomehine protons at δ_H_ 4.09 (1H, *br d*, *J* = 3.4) and δ_H_ 4.66 (1H, *dd*, *J* = 7.5 and 2.7 Hz), an ABX spin system at δ_H_ 5.17 (1H, *d*, *J* = 11.0 Hz), δ_H_ 5.49 (1H, *d*, *J* = 17.0 Hz) and δ_H_ 6.29 (1H, *dd*, *J* = 17.0, 11.0 Hz), and two terminal olefinic protons at δ_H_ 5.23 and 5.13 (each 1H, s). The COSY spectrum exhibited four spin systems coupled with ring A, ring B, and the side chain (Figure 2). All these accumulated data are regular with the plain skeleton of *neo*-clerodane diterpenes formerly isolated from this genus [27,40,55].

Interpretation of the 2D NMR data, including DQF-COSY, HMQC and HMBC, clearly indicated that we are dealing with a structure similar to that of stachaegyptin A (**5**), previously isolated from this species, and its structure was confirmed by X-ray crystallography [40]. The distinct difference observed in the ^1^H NMR spectrum of **1** was the additional oxymethine proton at δ_H_ 4.66 (1H, *dd*, *J* = 7.5 and 2.7 Hz) (H-12), which showed couplings in the DQF-COSY spectrum with H_2_-11 at δ_H_ 1.64 (1H, *dd*, *J* = 16.5, 7.5 Hz) (H-11a) and δ_H_ 1.50 (1H, *dd*, *J* = 16.5, 2.7 Hz) (H-11b), while in the HMQC spectrum this proton showed a correlation with the oxymethine carbon at δ_C_ 83.7. The ^13^C NMR data of **1** also revealed similarities with those of stachaegyptin A (**5**) except that the methylene carbon C-12 in **5** was replaced by the oxomethine carbon at δ_C_ 83.7 in **1**. The HMBC experiment (Figure 2) confirmed the presence of 12-oxymethine in **1** by the HMBC connections from H-12 (δ_H_ 4.66) to C-9 (δ_C_ 39.6), C-11 (δ_C_ 41.2), C-14 (δ_C_ 134.8) and C-16 (δ_C_ 116.5). With four oxygen atoms in **1** (C_20_H_30_O_4_, HR-FAB-MS), three of them were assigned from the ^13^C NMR data as two oxomethine carbons [δ_C_ 73.2 (C-7) and δ_C_ 83.7 (C-12)] and one keto group at δ_C_ 199.8 (C-2). Additionally, and due to the lack of an additional oxymethine signal, the remaining oxygen should, therefore, be a part of a hydroperoxyl group instead of a hydroxyl group.

This was supported by the positive TLC spray test for hydroperoxides (*N*,*N*-dimethyl-1,4-phenylenediammonium chloride) [56] as well as from the unusual downfield chemical shift of 12-oxymethine at δ_C_ 83.6, which was very similar to those reported for related 12-hydroperoxy diterpenes [56,57]. Related 12-hydroxy diterpenes, by contrast, showed a 12-oxymethine between δ_C_ 62.0–64.0 [58,59,60]. Comprehensive assignment of **1** was established from the results of DQF-COSY, HMQC, and HMBC NMR experiments. Therefore, **1** could be elucidated as 12-hydroperoxy derivative of **5**.

**Figure 3:**
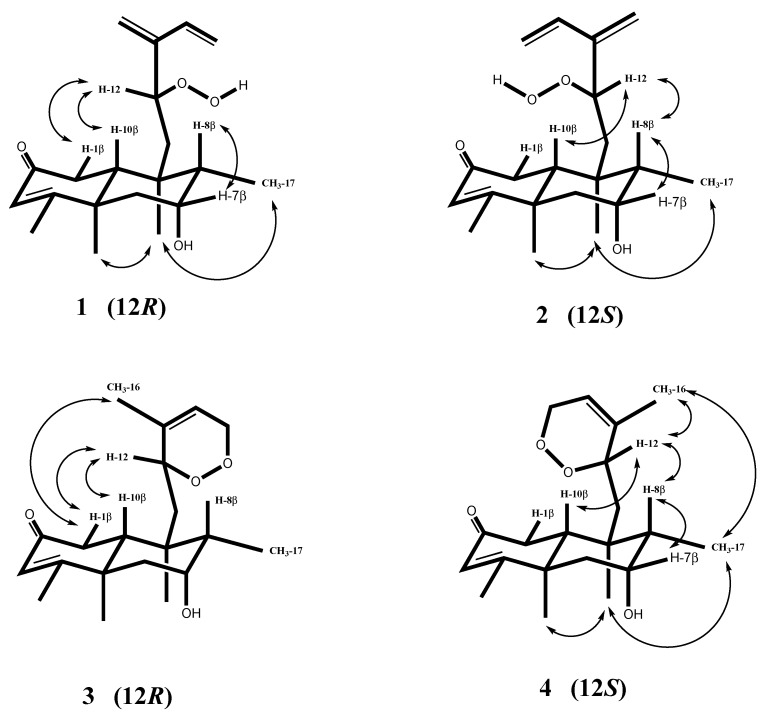
Stereo configurations based on NOESY correlations and 3D molecular model for **1**–**4.**

The relative stereochemistry of **1** was determined by the coupling constants, the NOESY experiments (Figure 3) with inspection of the 3D molecular model, and the biogenetic correlation with stachaegyptin A (**5**), where its structure and stereochemistry were confirmed by X-ray crystallography [40]. The hydroxyl group configuration at C-7 was assigned to be α (axial), conferring the small coupling constants of H-7 (3.4 Hz), which was similar to those reported for **5** and other *neo*-clerodane diterpenes [27,40]. The NOESY connections between H-7 (δ_H_ 4.09) and H-8 (δ_H_ 1.90) indicated that these protons are on *β*-configuration of the B ring. The NOESY correlations observed between CH_3_-17 (δ_H_ 1.09) and CH_3_-20 (δ_H_ 1.02) and between CH_3_-20 and CH_3_-19 (δ_H_ 1.39) indicated that these methyl groups are all on the same side in an *α*-configuration. The absence of a NOESY correlation between CH_3_-19α and H-10 revealed that the A/B ring system was trans-diaxially oriented, and the orientation of H-10 was β. All of previous results were well matched with the biogenetic precedent and formerly reported NMR chemical shift data for stachaegyptin **5** and related *neo*-clerodane diterpenes with the same configurations [27,40]. The C-12 configuration was determined by the NOESY analysis with inspection of the 3D molecular model (Figure 3). The observed correlations between H-12 (δ_H_ 4.66), H-1β (δ_H_ 2.29), and H-10 (δ_H_ 2.14) implied that these protons were in closeness and confirmed that the C-12 stereo center had the *R* configuration as those reported for (12*R*) 12-hydroperoxy and 12-hydroxy diterpenes [56,57,58,59,60,61,62]. Therefore, the structure of **1** was established as 12(*R*)-12-hydroperoxy-7α-hydroxy-*neo*-cleroda-3,13(16),14-triene-2-one, and was named stachaegyptin F.

Compound **2** was isolated as a colorless oil with an optical rotation of ${\left[\alpha \right]}_{D}^{25}$29 (c, 0.005, MeOH). The FAB-MS spectrum of **2** exhibited the base peak at *m/z* 357 [M + Na]^+^, consistent with a molecular formula C_20_H_30_O_4_, which was established by a molecular ion peak at *m/z* 357.2042[M + Na]^+^ (calcd. for C_20_H_30_O_4_Na, 357.2044) in the HR-FAB-MS analysis. This formula was the same as that reported for **1**. The positive reaction on TLC with *N*,*N*-dimethyl-1,4-phenylenediammonium chloride) [60] also revealed the presence of a hydroperoxid as in **1**. The ^1^H and ^13^C NMR spectra of **2** (Table 1 and Table 2) were almost identical with those reported for **1**, except for the upfield chemical shifts of CH_3_-17 (δ_H_ 0.99) as well as H-8 (δ_H_ 1.71), in addition to the downfield shift of H-1β (δ_H_ 2.60) in **2** comparing with those of **1.** The 2D NMR experiments including the DQF-COSY, HMQC, and HMBC exhibited an identical planar structure to that of **1**. Additionally, combined NOESY and coupling contacts analysis clearly indicated that **2** is matching the relative stereochemistry of **1** in the bicyclic system. All the above data and differences between **1** and **2** established that **2** should be an epimer of **1** at C-12 (*S* configuration) as previously shown in related compounds [57,60,61,62]. This was supported by the NOESY experiment with inspection of the 3D-molecular model (Figure 3). The strong correlations between H-12, H-10β, and H-8β, together with the absence of a NOESY correlation between H-12 and H-1β, confirmed the *S* configuration at C-12 in **2** instead of 12*R* as in **1**.

Further confirmation was given by the relative downfield shift of H-1β at δ_H_ 2.60 in **2**, instead of that at δ_H_ 2.29 in **1**, which was attributed to the presence of H-1β in a close proximity to the hydroperoxyl group. By contrast, H-8β and CH_3_-17 were slightly shifted at higher-field (δ_H_ 1.71 and δ_H_ 0.99, respectively), than those of **1** at δ_H_ 1.90 (H-8β) and δ_H_ 1.09 (CH_3_-17) [57,59,61,62]. Accordingly, the structure of **2** was established as 12(*S*)-12-hydroperoxy-7α-hydroxy-*neo*-cleroda-3,13(16),14-triene-2-one, and was named stachaegyptin G. Both epimers **1** and **2** have 6 stereocenters, and only one center (C-12) was inverted from 12*R* to 12*S*. Therefore, **1** and **2** are diastereomers.

Compound **4** was isolated as a colorless oil with an optical rotation of ${\left[\alpha \right]}_{D}^{25}$-10 (c, 0.005, MeOH). The molecular formula C_20_H_30_O_4_ was recognized from the HR-FAB-MS analysis, which exhibited a molecular ion peak at *m*/*z* 357.2044 [M + Na]^+^ (calcd. for C_20_H_31_O_4_Na, 357.2042), demonstrating six degrees of unsaturation in agreement with the ^13^C NMR spectrum of **4** (Table 2), which displayed 20 carbon resonances. Their multiplicities were determined from DEPT analysis as five methyls, four methylenes (one oxygenated at δ_C_ 69.8), six methines (two olefinic and two oxygenated at δ 73.3 and 79.0), and five quaternary carbons (two olefinic and one keto at δ 200.7). The ^1^H NMR spectrum of **1** (Table 1) exhibited characteristic signals for two tertiary methyls at δ_H_ 1.07 and 1.39 (each 3H, *s*), a secondary methyl at δ_H_ 1.06 (3H, *d*, *J* = 7.0 Hz), and two olefinic methyls at δ_H_ 1.71 and 1.88 (each 3H, *s*), which showed correlations in the DQF-COSY spectrum with two olefinic protons at δ_H_ 5.57 (1H, *d*, *J* = 2.5 Hz) and 5.69 (1H, *br s*), respectively, indicating the presence of two trisubstituted double bonds. The spectrum also showed two oxomehine protons at δ_H_ 4.11 (1H, *br d*, *J* =2.7) and δ_H_ 4.18 (1H, *br d*, *J* = 10.3 Hz), as well as two protons of an oxymethylen at δ_H_ 4.61 (1H *br dd*, *J* = 16.5, 10.3) and δ_H_ 4.29 (1H, *br d*, *J* = 14.4 Hz). The COSY spectrum exhibited four spin systems associated with ring A, ring B, and the side chain (Figure 2).

The ^1^H and ^13^C NMR spectra as well as the 2D NMR data, including DQF-COSY, HMQC and HMBC (Figure 2), clearly established that we are dealing with a structure almost identical to that of stachyaegyptin C (**3**), previously isolated from this species [41]. The distinct differences observed in the ^1^H NMR spectrum of **4** showed a slightly higher-field position chemical shift of CH_3_-17 (δ_H_ 1.06) in **4** than that in **3** (δ_H_ 1.13), also H-8 was shifted at higher field (δ_H_ 1.69) in **4** than that of **3** (δ_H_ 2.06). In contrary, the chemical shift of H-1β was at lower field value (δ_H_ 2.80) in **4** than **3** (δ_H_ 2.32). The results of the 2D NMR experiments achieved an indistinguishable planar structure to that of **3**. The NOESY and coupling contacts analysis clearly indicated that **4** had identical relative stereochemistry with **3** in the bicyclic system. All the above data and differences between **4** and **3** established that compound **4** should be an isomer of **3** epimerized at C-12 (*S* configuration). This result was supported by the NOESY experiment with inspection of the 3D molecular model (Figure 3).

The strong correlations of H-12 with H-10β, H-8β, and CH_3_-16, and the correlation between CH_3_-17 with H-11a (1.42) and CH_3_-16, as well as the absence of a NOESY correlation between H-12 and H-1β, confirmed the *S* configuration at C-12 instead of 12*R* in **3**. Further confirmation was given by the relative downfield shift of H-1β at δ_H_ 2.80 in **4**, instead of that at δ_H_ 2.11 in **3**, which was attributed to the presence of H-1β in a close proximity to the cyclic peroxide ring. On the other hand, H-8β and CH_3_-17 were slightly shifted at higher field (δ_H_ 1.69 and δ_H_ 1.06, respectively) than those of **3** at δ_H_ 2.32 (H-8β) and δ_H_ 1.13 (CH_3_-17) [61,63,64,65,66]. Accordingly, the structure of **4** was established as 12(*S*)-12,15-peroxy-7α-hydroxy-*neo*-cleroda-3,13-diene-2-one, and was named as stachaegyptin H. Compounds **3** and **4** have 6 stereocenters, and only one center (C-12) was inverted from 12*R* to 12*S*. Accordingly, **3** and **4** are diastereomers.

To the best of our knowledge, these new diterpenes hydroperoxides (**1** and **2**) and the cyclic peroxide (**4**) are rare secondary metabolites.

## 3. Proposed Biosynthetic Pathway of the Isolated Compounds

**Figure 4:**
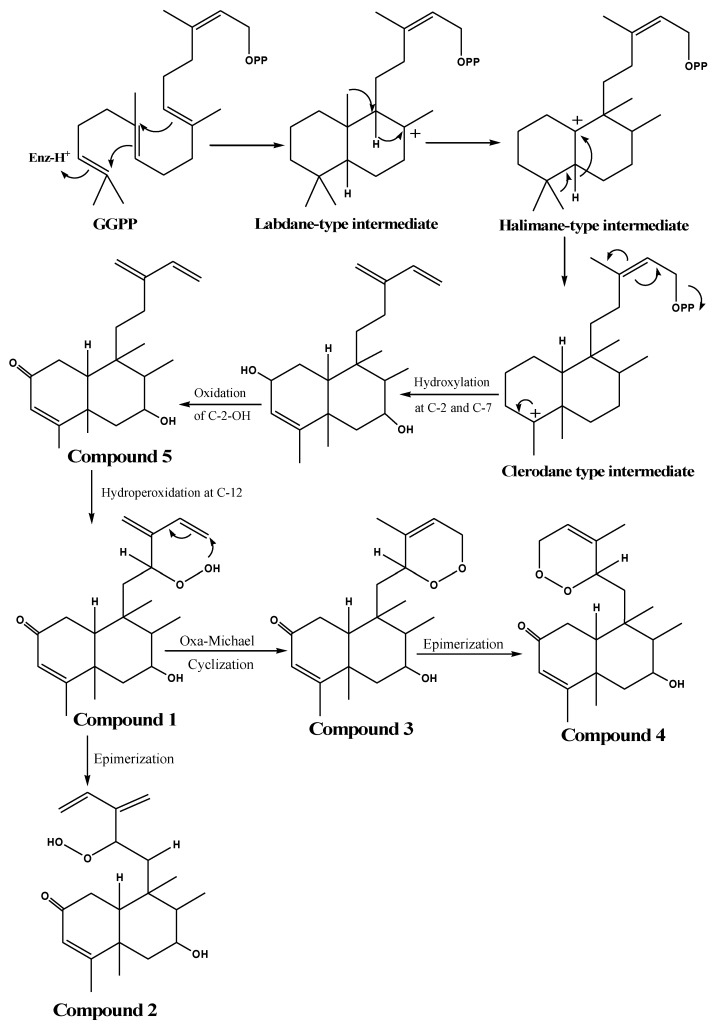
Proposed scheme for the biosynthesis pathway of the isolated metabolites (**1**–**5**).

Biosynthetically, diterpenoids classes in plant catalyze a proton-initiated cationic cycloisomerization of geranylgeranyl diphosphate (GGPP), generating a labdane-type intermediate [63]. Subsequently, labdane as precursor can undergo a stepwise migration process of methyl and hydride shift, yielding a halimane-type intermediate, which can then progress to either *cis* or *trans* clerodanes [31]**.** Compound **5** is proposed to go through simply enzymatic hydroxylation and oxidation of clerodane-type intermediate [64]. Based on Capon’s model for biosynthesis of endoperoxides, compound **5** is subjected to enzymatic hydroperoxidation at C-12 to generate compound **1,** which then undergoes oxa-Michael cyclization to produce compound **3** [65]. In addition, both compound **1** and **3** can generate their corresponding epimers **2** and **4**, respectively, by further rearrangement and isomerization reactions (Figure 4).

## 4. Chemosystematic Significance

Different diterpenoids types of *ent*-clerodane, kaurane, labdane, and rosane were isolated from about 27 species of *Stachys* including the present one that is known to produce around 35 compounds/classes of terpenes. The kaurane, labdane, *ent*-labdane, and rosane types of diterpenoids were rare, while only the *neo*-clerodane ones were common. The 2,7 di-substituted *neo*-clerodane derivatives were reported as annuanone, which was isolated from three species, *S. annua*, *S. inflate,* and *S. Sylvatica* [66]; stachysolone from *S. recta* [37], *S. annua* [66], and *S. lavandulifolia* [67]; 7-mono-acetyl-stachysolone in *S. recta* [37] and *S. annua* [66]; diacetyl-stachysolone from *S. aegyptiaca* [41]; stachone and stachylone in *S. inflate*, *S. atherocalyx*, *S. annua,* and *S. palustris* [66]. The 2,3,4 tri-substituted *neo*-clerodane as reseostetrol was isolated from *S. rosea* [68] and 3α,4α-epoxy rosestachenol from in *S. glutinosa* besides the mono-substituted neo-clerodanes as roseostachone and roseostachenol in *S. rosea* [55]. However, the kaurane-type diterpenoids were represented only in peroxide form as stachyperoxide from *S. aegyptiaca* [41].

In addition, four hydroxylated kaurane derivatives, i.e., 3α,19-dihydroxy-*ent*-kaur-16-ene, 3α-hydroxyl-19-kaur-16-en-oic acid from *S. lanata,* and 6β-hydroxyl-*ent*-kaur-16-ene, and 6β,18-dihydroxy *ent*-kaur-16-ene from *S. sylvatica* [64] were isolated. Rare labdane diterpenoids were found only in one species as (+)-13-*epi*-Jabugodiol, (+)-6-deoxy-andalusol, and (+)-plumosol from *S. plumose* [42]. Also, only two *ent*-labdane diterpenoids, namely ribenone and ribenol in *S. mucronata* [39], as well as only three rosane diterpenoids, were reported from *S. paraviflora* as stachyrosane, stachyrosane 1, and 2 [38,69]. In the present study, five *neo*-clerodane diterpenoids including four *ent*-*neo*-clerodane peroxides were isolated from *S. aegyptiaca*. The comparative study of previous data revealed that *S. aegyptiaca* is characterized by having the capability to produce *neo*-clerodane peroxides, which are different than other reported diterpenoids from other *Stachys* species. This proved that the *S. aegyptiaca* has a unique biosynthetic pathway to generate *neo*-clerodane peroxides recognized as rare types of clerodanes. Those are known for their significant biological activities as anticancer, antimitotic, and antifungal [70,71] and used in treatment of various inflammation and metabolic disorders [72].

## 5. Materials and Methods

### 5.1. General Procedures

The ^1^H NMR (600 MHz, CDCl_3_), ^13^C NMR (150 MHz, CDCl_3_), and the 2D NMR spectra were recorded on a JEOL JNM-ECA 600 spectrometer (JEOL Ltd., Tokyo, Japan). All chemical shifts (δ) are given in ppm units with reference to TMS as an internal standard, and coupling constants (J) are reported in Hz. The IR spectra were taken on a Shimadzu FT-IR-8100 spectrometer. Specific rotations were measured on a Horiba SEPA-300 digital polarimeter (l = 5 cm). FAB-MS and HR-FAB-MS were recorded on a JEOL JMS-GC-MATE mass spectrometer. For chromatographic separations COSMOSIL-Pack type (C18-MS-II) (Inc., Cambridge, MA 02138, USA, 250 × 4.6 mm i.d.) and (250 × 20 mm i.d.) columns were used for analytical and preparative separations, respectively, with compound detection via a Shimadzu RID-10 A refractive index detector. For open silica gel column separations, normal-phase column chromatography employed BW-200 (Fuji Silysia, Aichi, Japan, 150–350 mesh) and reversed-phase column chromatography employed Chromatorex ODS DM1020 T (Fuji Silysia, Aichi, Japan, 100–200 mesh). TLC separations used precoated plates with silica gel 60 F_254_ (Merck, Pfizer, Sanofi, 0.25 mm) (ordinary phase) or reversed-phase precoated plates with silica gel RP-18 WF254S (Merck, Pfizer, Sanofi, 0.25 mm) with compounds observed by spraying with H_2_SO_4_-MeOH (1:9) followed by heating.

### 5.2. Plant Material

The aerial parts of *S. aegyptiaca* were collected from Southern Sinai in Egypt during May 2016. A voucher specimen (SK-1055) has been deposited in the Herbarium of Saint Katherine protectorate, Egypt, with collection permission granted for scientific purposes by the Saint Katherine protectorate.

### 5.3. Extraction and Isolation

Extraction and fractionation of the air-dried aerial parts of *S. aegyptiaca* (1.5 kg) were previously described [40]. The *n*-hexane-CH_2_Cl_2_ (1:3) fraction (14.0 g) and 100% CH_2_Cl_2_ (7.0 g) were added together due to same chromatographic system then chromatographed on a ODS column (3 × 90 cm) eluted with 80%, 90% (MeOH:H_2_O) then washed with 100% MeOH. Fractions were obtained as two main portions: A (6.0 g) and B (7.0 g). Subfraction A was re-purified by reversed-phase HPLC using MeOH/H_2_O (65–35% 500 mL) to afford **5** (20 mg). Subfraction B was re-purified by reversed-phase HPLC using MeOH:H_2_O (70:30%, 1000 mL) to afford **3** (10 mg) and **4** (12 mg). The 5% MeOH fraction (8.5 g) was chromatographed on ODS column (3 × 90 cm) eluted with 80%, 90% (MeOH:H_2_O) then washed with MeOH. Fractions were obtained as one main portion (2.5 g), which was re-purified by reversed-phase HPLC using MeOH:H_2_O (80:20%, 1000 mL) to afford **2** (9 mg) and **3** (11 mg).

The 12(*R*)-12-hydroperoxy-7α-hydroxy-neo-cleroda-3,13(16),14-triene-2-one (stachaegyptin F, **1**). Colorless oil, ${\left[\alpha \right]}_{D}^{25}$+30 (c, 0.001, MeOH), ^1^H (CDCl_3_, 600 MHz), and ^13^C (CDCl_3_, 150 MHz) NMR, see Table 1 and Table 2; FAB-MS *m*/*z* 335 [M + H]^+^ HR-FAB-MS *m*/*z* 357.2045 (calcd. for C_20_H_30_O_4_Na, 357.2044); IR (ν_max_ cm^−^^1^): 3445, 1665 and 1615 cm^−1^.

The 12(*S*)-12-Hydroperoxy-7α-Hydroxy-neo-cleroda-3,13(16),14-triene-2-one (stachaegyptin G, **2**). Colorless oil, ${\left[\alpha \right]}_{D}^{25}$-29 (c, 0.005, MeOH), ^1^H (CDCl_3_, 600 MHz), and ^13^C (CDCl_3_, 150 MHz) NMR, see Table 1 and Table 2; FAB-MS *m/z* 335 [M + H]^+^ HR-FAB-MS *m*/*z* 357.2042 (calcd. for C_20_H_30_O_4_, 357.2044); and *m/z* 357.2044 (calcd. for C_20_H_30_O_4_Na, 335.2042); IR (ν_max_ cm^−^^1^): 3445, 1665, and 1615 cm^−1^.

The 12(*S*)-12,15-peroxy-7α-Hydroxy-neo-cleroda-3,13-diene-2-one (stachaegyptin H, **4**).

Colorless oil, ${\left[\alpha \right]}_{D}^{25}$-10 (c, 0.005, MeOH), ^1^H (CDCl_3_, 600 MHz), and ^13^C (CDCl_3_, 150 MHz) NMR, see Table 1 and Table 2; FAB-MS *m/z* 335 [M + H]^+^ HR-FAB-MS *m*/*z* 357.2044 (calcd. for C_20_H_30_O_4_Na, 357.2042); IR (ν_max_ cm^−1^): 3450, 1660, and 1620 cm^−1^.

## Figures and Tables

**Table 1:** The ^1^H NMR data assignments for compounds **1**–**4** (600 MHz, in CDCl_3_) ^a^.

Position	1	2	3 ^a^	4
1α	2.41 dd, (17.0, 14.0)	2.41 dd (17.0, 14.4)	2.52 dd (17.0, 14.0)	2.41 m *
1β	2.29 dd (17.0, 3.4)	2.60 dd (17.0, 2.8)	2.32 dd (17.0, 3.4)	2.80 dd (17.0, 3.4)
2	---	---	---	---
3	5.68 br s	5.68 br s	5.69 br s	5.69 br s
4	---	---	---	---
5	---	---	---	---
6α	2.20 dd (14.0, 2.7)	2.22 dd (14.0, 2.7)	2.19 dd (14.0, 2.7)	2.17 dd (14.0, 2.7)
6β	1.60 dd (14.0, 3.4)	1.63 dd (14.0, 3.4)	1.57 dd (14.0, 3.4)	1.57 dd (14.0, 3.4)
7	4.09 br d (3.4)	4.11 br d (2.4)	4.07 m	4.11 br d (2.7)
8	1.90 m *	1.71 m	2.06 m	1.69 m *
9	---	---	---	---
10	2.14 dd (14.0, 3.4)	2.25 dd (14.0, 2.8)	2.11 dd (14.0, 3.4)	2.41 m *
11a	1.64 dd (16.5, 7.5)	1.62 dd (16.5, 7.5)	1.91 dd (14.0, 10.5)	1.96 dd (16.5, 10.3)
11b	1.50 dd (16.5, 2.0)	1.52 dd (16.5, 2.0)	1.44 m *	1.42 m *
12	4.66 dd (7.5, 2.7)	4.66 d (8.2)	4.18 br d (10.5)	4.18 d (10.3)
13	---	---	---	---
14	6.29 dd (17.0, 11.0)	6.31 dd (17.0, 11.0)	5.58 br s	5.57 br d (2.5)
15a	5.49 d (17.0)	5.45 d (17.0)	4.61 br d (14.0)	4.61 br dd (14.4, 2.5)
15b	5.17 d (11.0)	5.15 d (11.0)	4.28 br d (14.0)	4.29 br d (14.4)
16a	5.23 s	5.23 s	1.73 s	1.71 s
16b	5.13 s	5.18 s	---	---
17	1.09 d (7.0)	0.99 d (7.5)	1.13 d (7.0)	1.06 d (7.0)
18	1.92 s	1.91 s	1.91 s	1.88 s
19	1.39 s	1.39 s	1.42 s	1.39 s
20	1.02 s	1.01 s	1.07 s	1.07 s

^a^ Data are given for comparison with the new compound **4.** * Overlapping signals.

**Table 2:** The ^13^C NMR data assignments for compounds **1**-**4** (150 MHz, in CDCl_3_) ^a^.

C	1	2		3 ^a^	4	
	δ_C_	δ_C_	DEPT	δ_C_	δ_C_	DEPT
1	35.3	35.5	CH_2_	35.4	35.3	CH_2_
2	199.8	200.9	C=O	199.8	200.7	C=O
3	125.1	125.2	CH	125.0	125.5	CH
4	172.9	172.7	C	173.1	172.2	C
5	39.6	39.0	C	38.8	38.8	C
6	41.9	42.0	CH_2_	41.2	41.4	CH_2_
7	73.2	73.2	CH	73.3	73.3	CH
8	39.8	39.6	CH	39.7	38.8	CH
9	39.6	39.5	C	39.6	39.2	C
10	46.4	46.6	CH	45.9	46.4	CH
11	41.2	41.3	CH_2_	38.0	38.0	CH_2_
12	83.7	82.6	CH	79.2	79.0	CH
13	146.3	146.9	C	134.7	134.2	C
14	134.8	135.3	CH	118.7	119.1	CH
15	116.4 *	115.5	CH_2_	69.9	69.8	CH_2_
16	116.5 *	115.6	CH_2_	19.1	19.0	CH_3_
17	12.8	12.7	CH_3_	12.5	12.6	CH_3_
18	19.4	19.2	CH_3_	19.7	19.4	CH_3_
19	20.2	20.4	CH_3_	20.3	20.6	CH_3_
20	19.1	19.1	CH_3_	19.4	19.3	CH_3_

^a^ Data are given for comparison with the new compound **4.** * Overlapping signals.

## Supplementary Materials

Supplementary data relating to this article is available online.
