# Supplementary material for: New Rare Ent-Clerodane Diterpene Peroxides from Egyptian Mountain Tea (Qourtom) and Its Chemosystem as Herbal Remedies and Phytonutrients Agents
Source: Molecules. 2020 May 6;25(9):2172. doi: 10.3390/molecules25092172 (PMC7248853; doi:10.3390/molecules25092172)
Supplement: Supplementary file 1 [file molecules-25-02172-s001.pdf]

Supplementary Materials

New rare *ent*-clerodane diterpene peroxides from Egyptian Mountain tea (Qourtom) and its chemosystematic as herbal remedies and phytonutrients agents.

Taha A. Hussien[1], Ahmed A. Mahmoud[2*], Naglaa S. Mohamed[3], Abdelaaty A. Shahat [4,5*], Hesham R. El-Seedi[6,7,8,9*], Mohamed Elamir F. Hegazy[5*]

[1] Pharmacognosy Department, Faculty of Pharmacy, Deraya University, Minia, Egypt; thussien71@yahoo.com (T.A.H.)

[2] Chemistry Department, Faculty of Science, Minia University, El-Minia 61519, Egypt; amahmoud@mu.edu.eg (A.A.M)

[3] Chemistry Department, Faculty of Science, Aswan University, Aswan 81528, Egypt; naglaanaglaa@yahoo.com (N.S.M.)

[4] Pharmacognosy Department, College of Pharmacy, King Saud University, P.O. Box 2457, Riyadh 11451, Saudi Arabia; alalqahtani@ksu.edu.sa (A.S.A.); ashahat@ksu.edu.sa (A.A.S.)

[5]Chemistry of Medicinal Plants Department, National Research Centre, 33 El-Bohouth St., Dokki, Giza, 12622, Egypt; ashahat@ksu.edu.sa (A.A.S.), elamir77@live.com (M.-E.F.H)

[6]The Wenner-Gren Institute, Stockholm University, S-106 91, Stockholm, Sweden

[7]International Research Center for Food Nutrition and Safety, Jiangsu University, Zhenjiang 212013, China

[8]Al-Rayan Research and Innovation Center, Al-Rayan Colleges, Medina 42541, Saudi Arabia

[9]Pharmacognosy Group, Department of Medicinal Chemistry, Uppsala University, Biomedical Centre, Box 574, 751 23 Uppsala, Sweden

* Correspondence: amahmoud@mu.edu.eg (A.A. Mahmoud); Tel: +2 01099338896; and hesham.elseedi@su.se (H.R. E-S) Tel.: +46 73 5668234; elamir77@live.com (M.E.F.Hegazy) Tel.: +2 033371635

Received: date; Accepted: date; Published: date

Received: date; Accepted: date; Published: date

Supporting data Page

S1:^{1}H NMR of **1**... 3

S2:^{13}C NMR of **1** 4

S3:DEPT-135 of **1**... 5

S4:^{1}H ^{1}H COSY of **1**.. 6

S5:HSQC of **1**... 7

S6:HMBC of **1**... 8

S7:NOESY of **1**... 9

S8:FABMS of **1**... 10

S9: HRFABMS of **1**.. 11

S10:^{1}H NMR of **2**.. 12

S11:^{13}C NMR of **2** 13

S12:DEPT-135 of **2**.. 14

S13:^{1}H ^{1}H COSY of **2**... 16

S14:HSQC of **2**.. 15

S15:HMBC of **2**.. 17

S16:NOESY of **2**.. 18

S17: FABMS of **2**.. 19

S18: HRFABMS of **2**... 20

S19:^{1}H NMR of **4**.. 21

S20:^{13}C NMR of **4** 22

S21:DEPT-135 of **4**.. 23

S22:^{1}H ^{1}H COSY of **4**... 24

S23:HSQC of **4**.. 25

S24:HMBC of**4**.. 26

S25:NOESY of **4**.. 27

S26: FABMS of **4**.. 28

S27: HRFABMS of **4**... 29

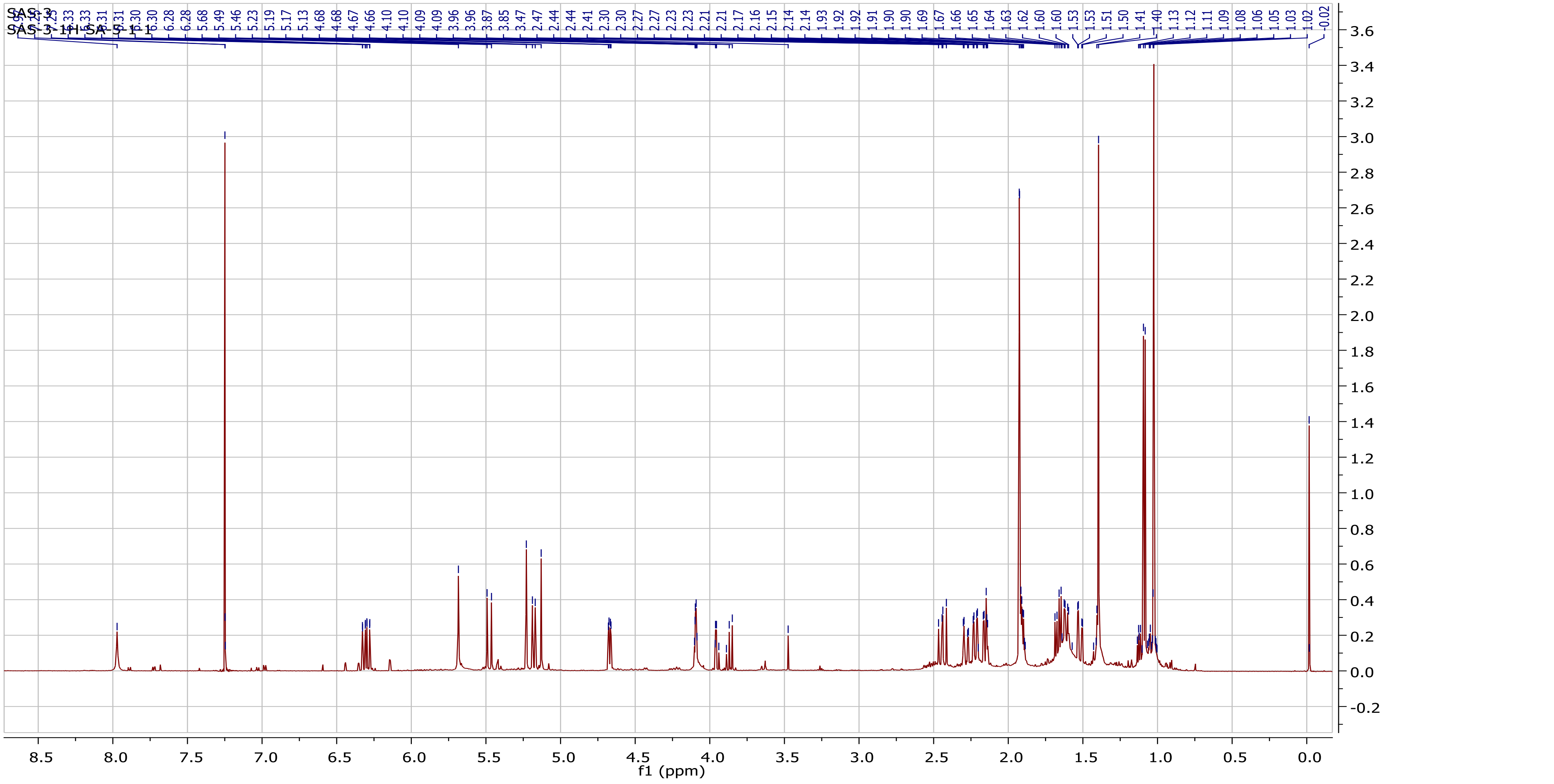

S1:^{1}H NMR of **1**

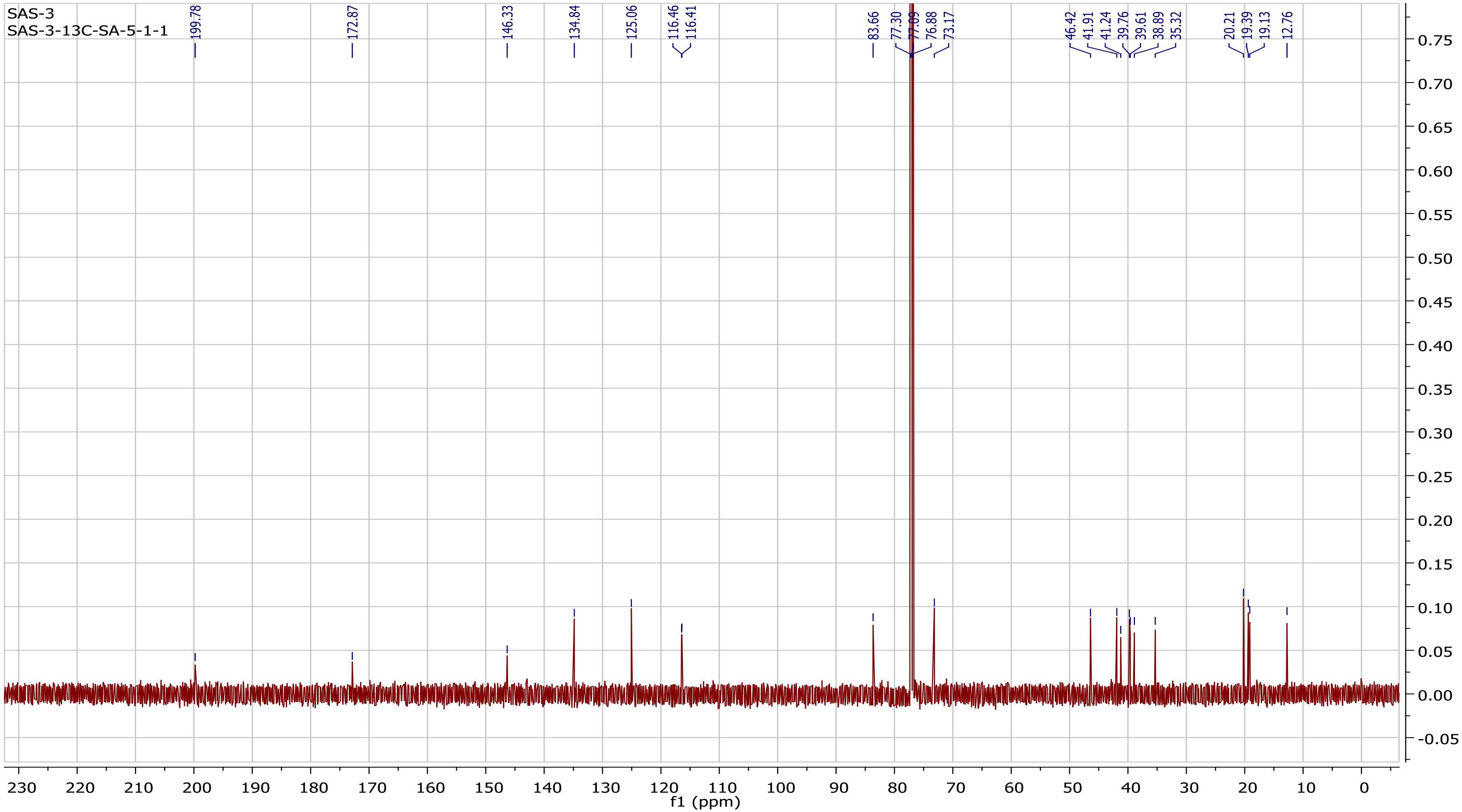

S2:^{13}C NMR of **1**

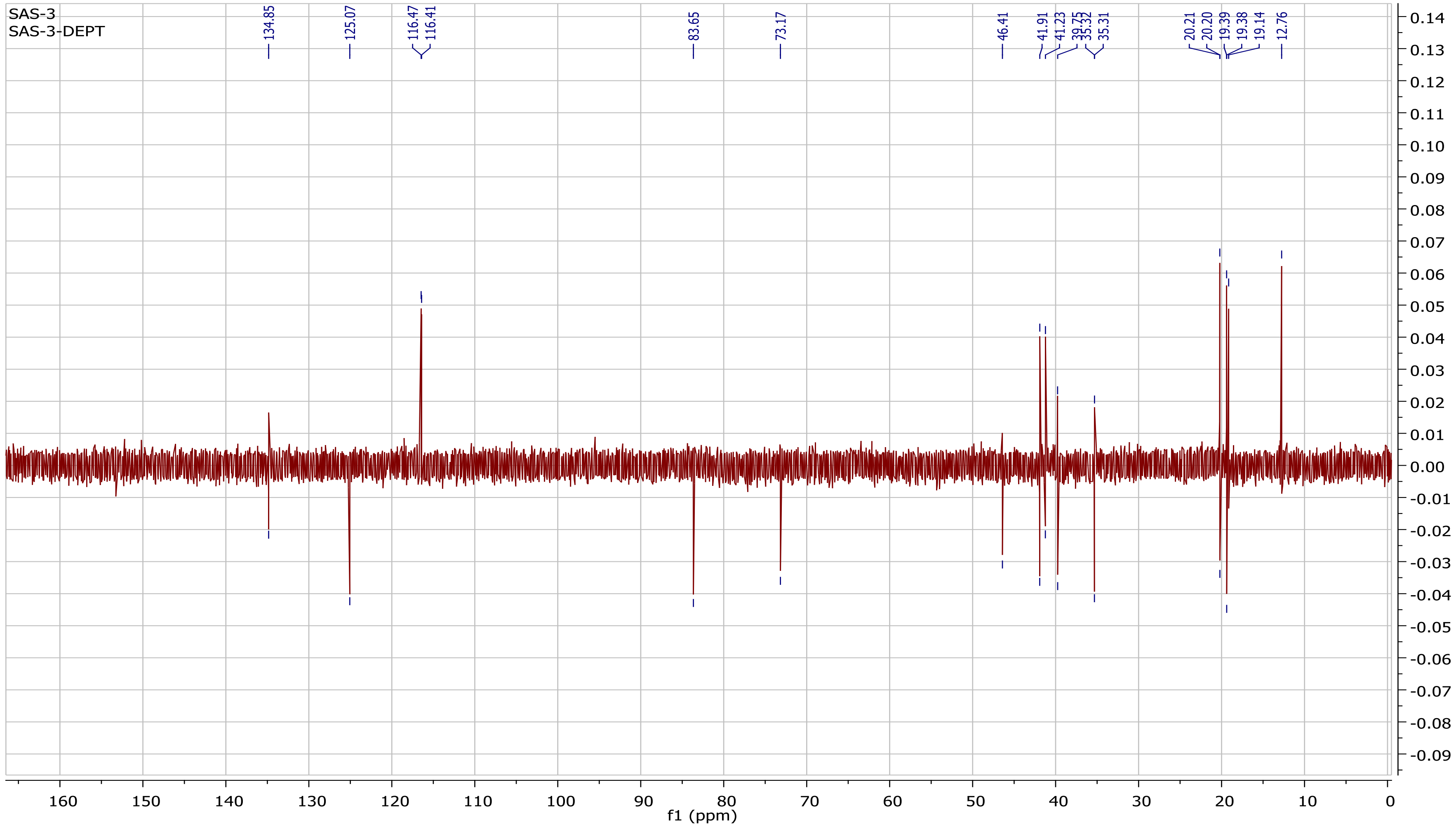

S3:DEPT-135 of **1**

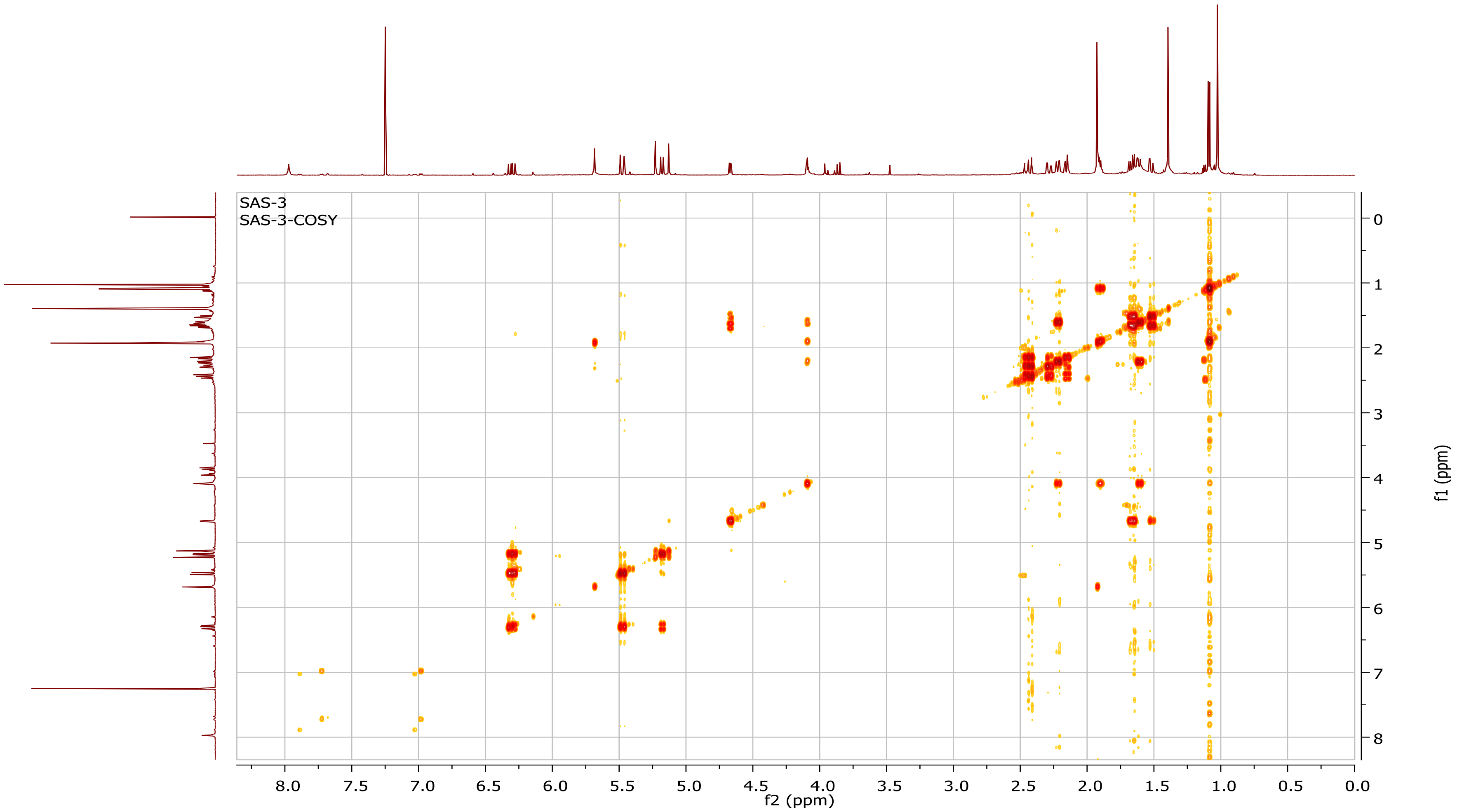

S4: ^{1}H ^{1}H COSY of **1**

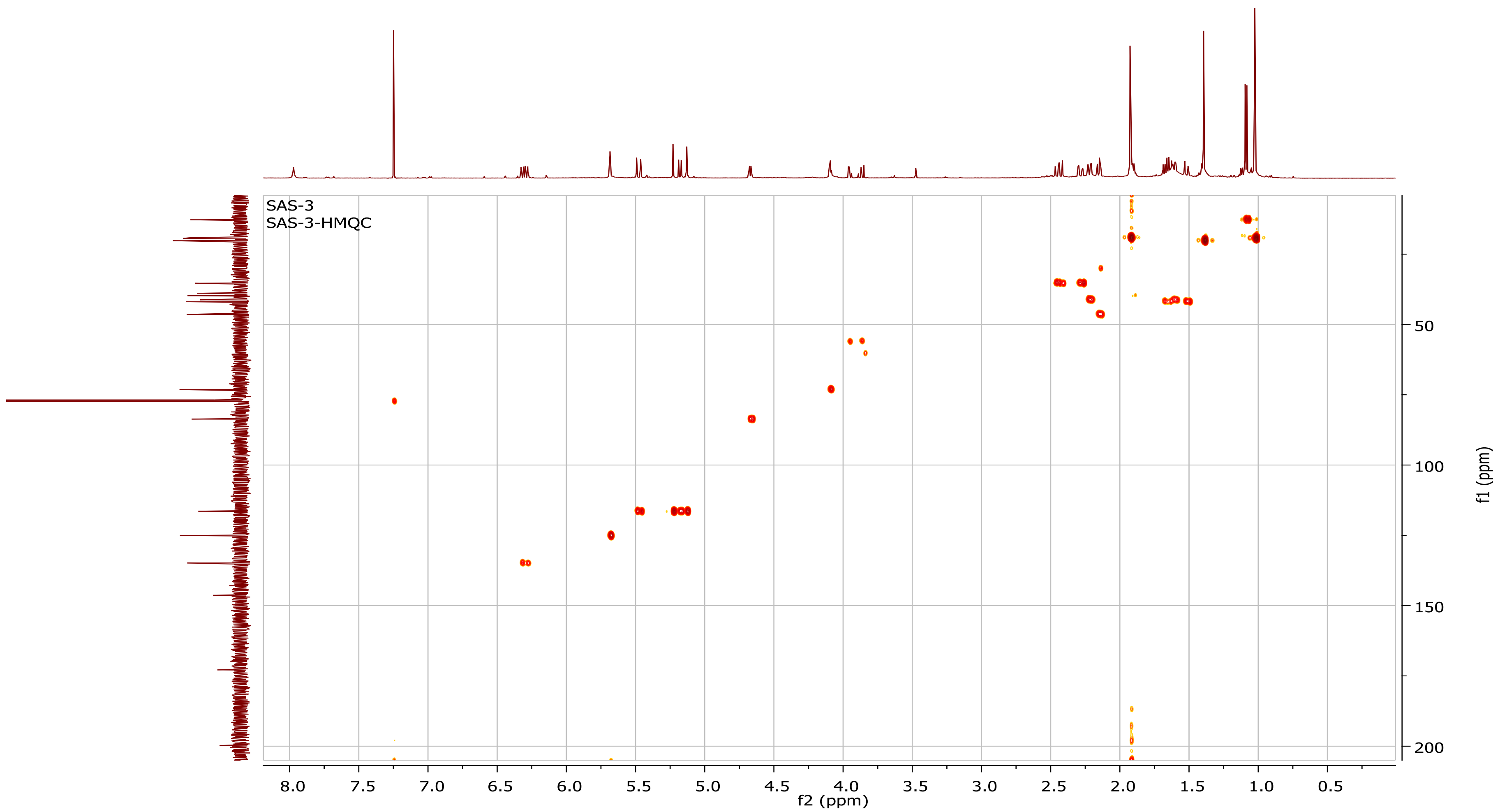
SAS-3
SAS-3-HMQC
8.0
7.5
7.0
6.5
6.0
5.5
5.0
4.5
4.0
3.5
3.0
2.5
2.0
1.5
1.0
0.5
f2 (ppm)
50
100
150
200
f1 (ppm)

S5:HSQC of **1**

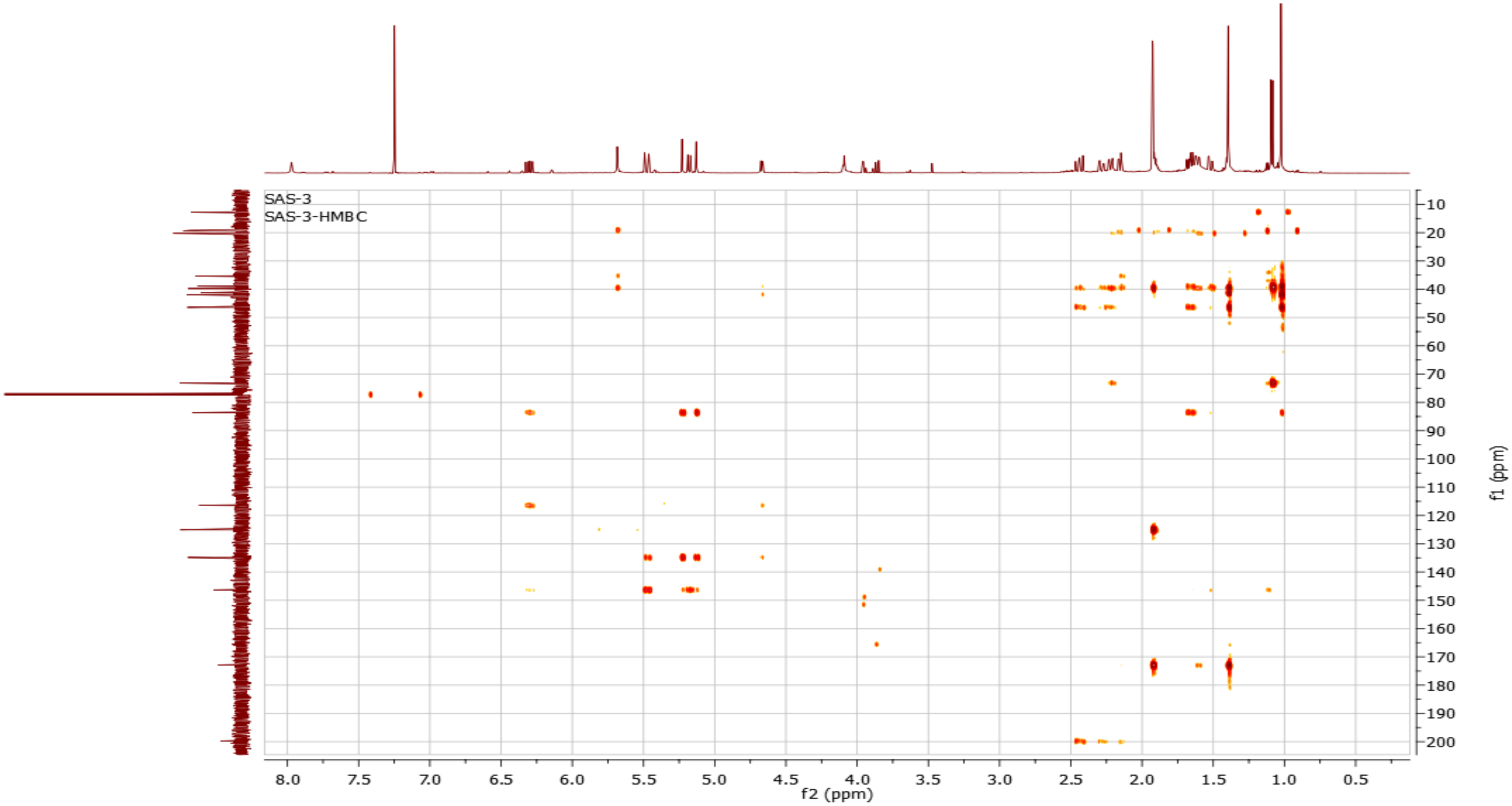

SAS-3
SAS-3-HMBC
8.0 7.5 7.0 6.5 6.0 5.5 5.0 4.5 4.0 3.5 3.0 2.5 2.0 1.5 1.0 0.5
f2 (ppm)
10 20 30 40 50 60 70 80 90 100 110 120 130 140 150 160 170 180 190 200
f1 (ppm)

S6:HMBC of **1**

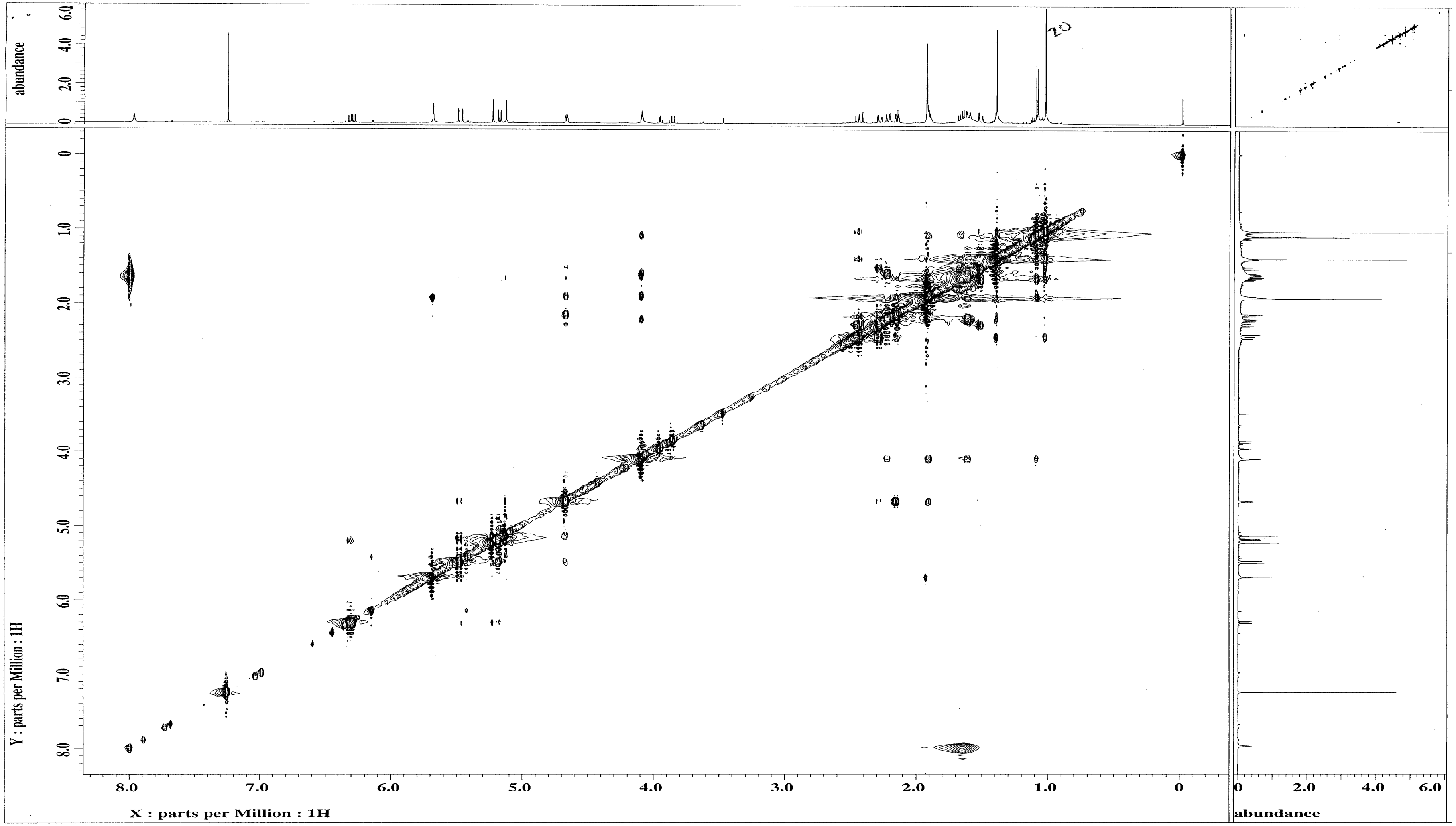
abundance
6.0
4.0
2.0
0
20
Y : parts per Million : 1H
8.0
7.0
6.0
5.0
4.0
3.0
2.0
1.0
0
X : parts per Million : 1H
abundance

S7:NOESY of **1**

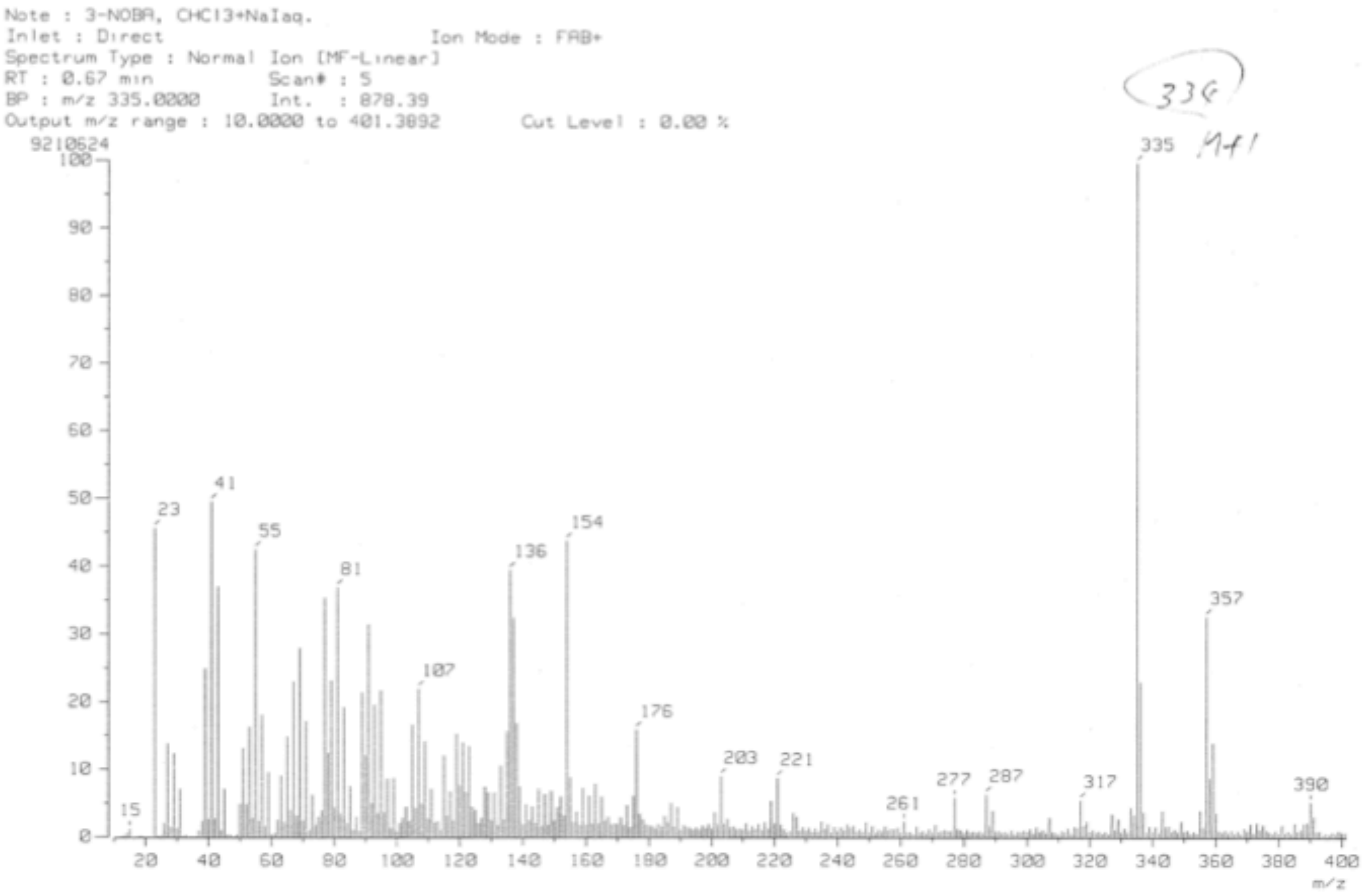
Note : 3-NOBA, CHCl3+NaIaq.
Inlet : Direct
Ion Mode : FAB+
Spectrum Type : Normal Ion [MF-Linear]
RT : 0.67 min
Scan# : 5
BP : m/z 335.0000
Int. : 878.39
Output m/z range : 10.0000 to 401.3892
Cut Level : 0.00 %
9210624
336
M+1
335
357
390
317
287
277
261
221
203
176
154
136
107
81
55
41
23
15
m/z

S8: FABMS of **1**

Note : 3-NOBA, CHCl3+NaIaq.
Inlet : Direct   Ion Mode : FAB+
RT : 0.90 min   Scan#: 4
Elements : C 20/0, H 40/0, O 4/0, Na 1/0
Mass Tolerance : 1000ppm, 3mmu if m/z < 3, 5mmu if m/z > 5
Unsaturation (U.S.) : -0.5 - 20.0

Observed m/z	Int%	Err[ppm / mmu]	U.S.	Composition
357.2045	16.7	+1.0 / +0.3	5.5	C 20 H 30 O 4 Na

S9:HRFABMS of **1**

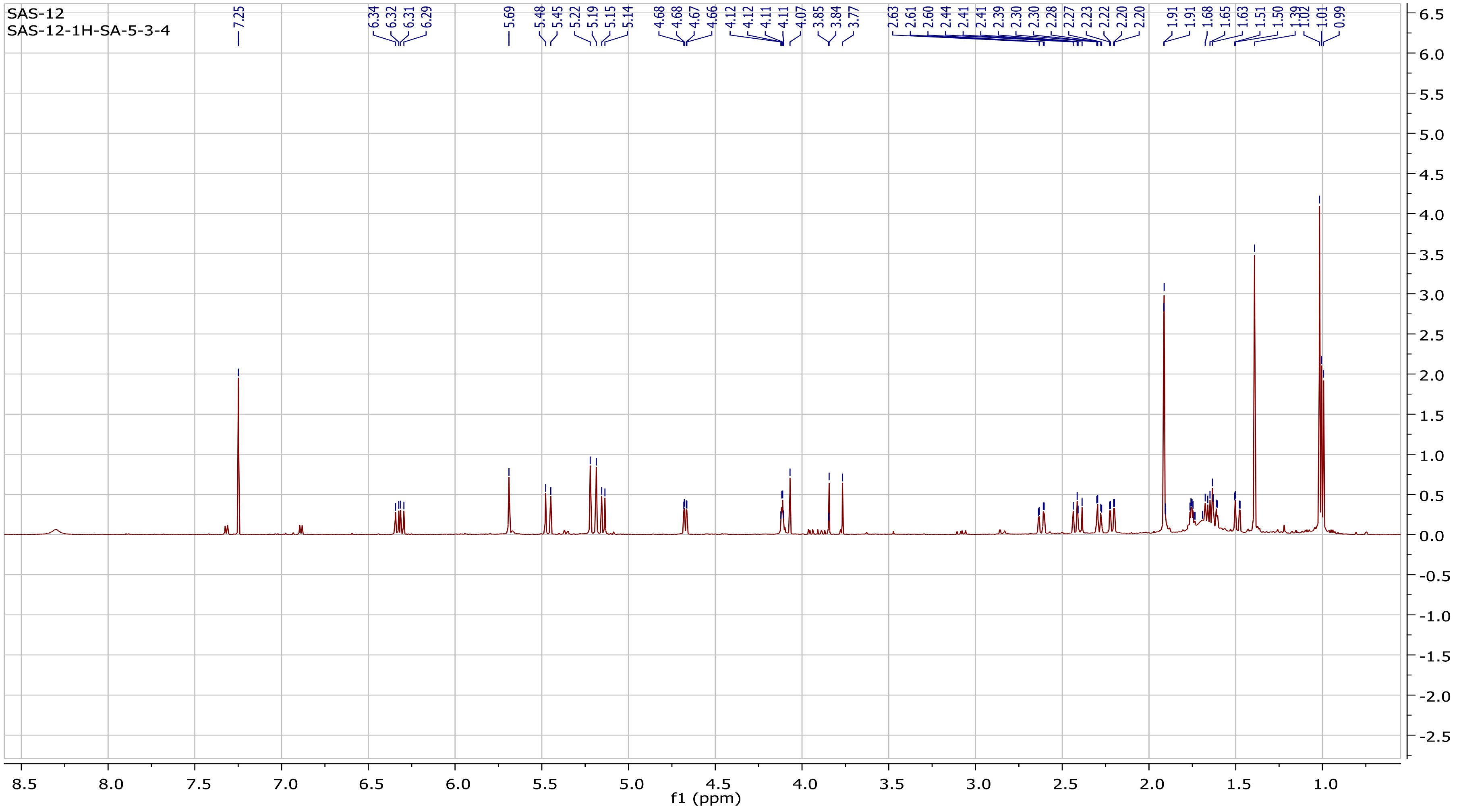

S10: ^{1}H NMR of **2**

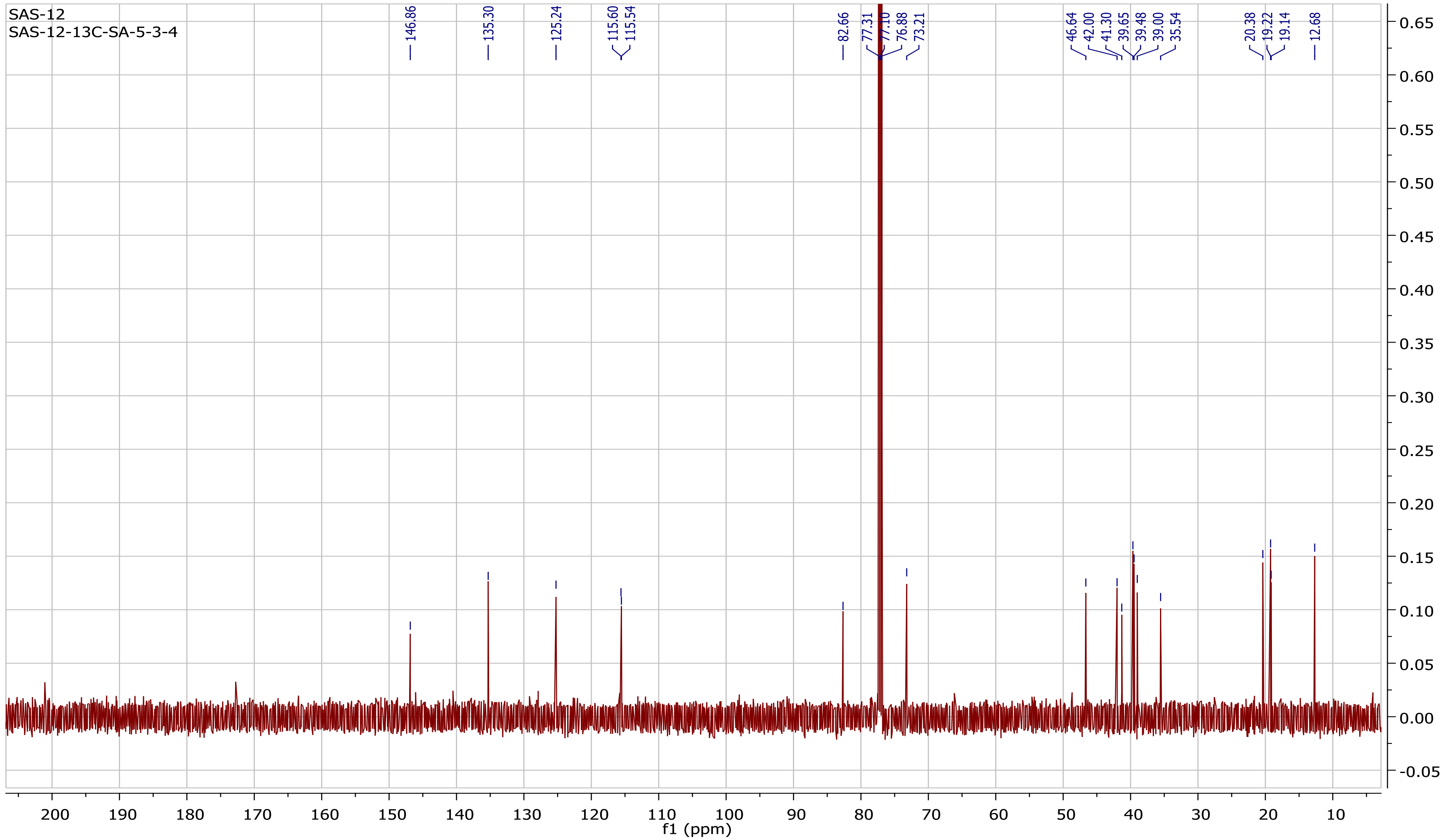

S11: ^{13}C NMR of **2**

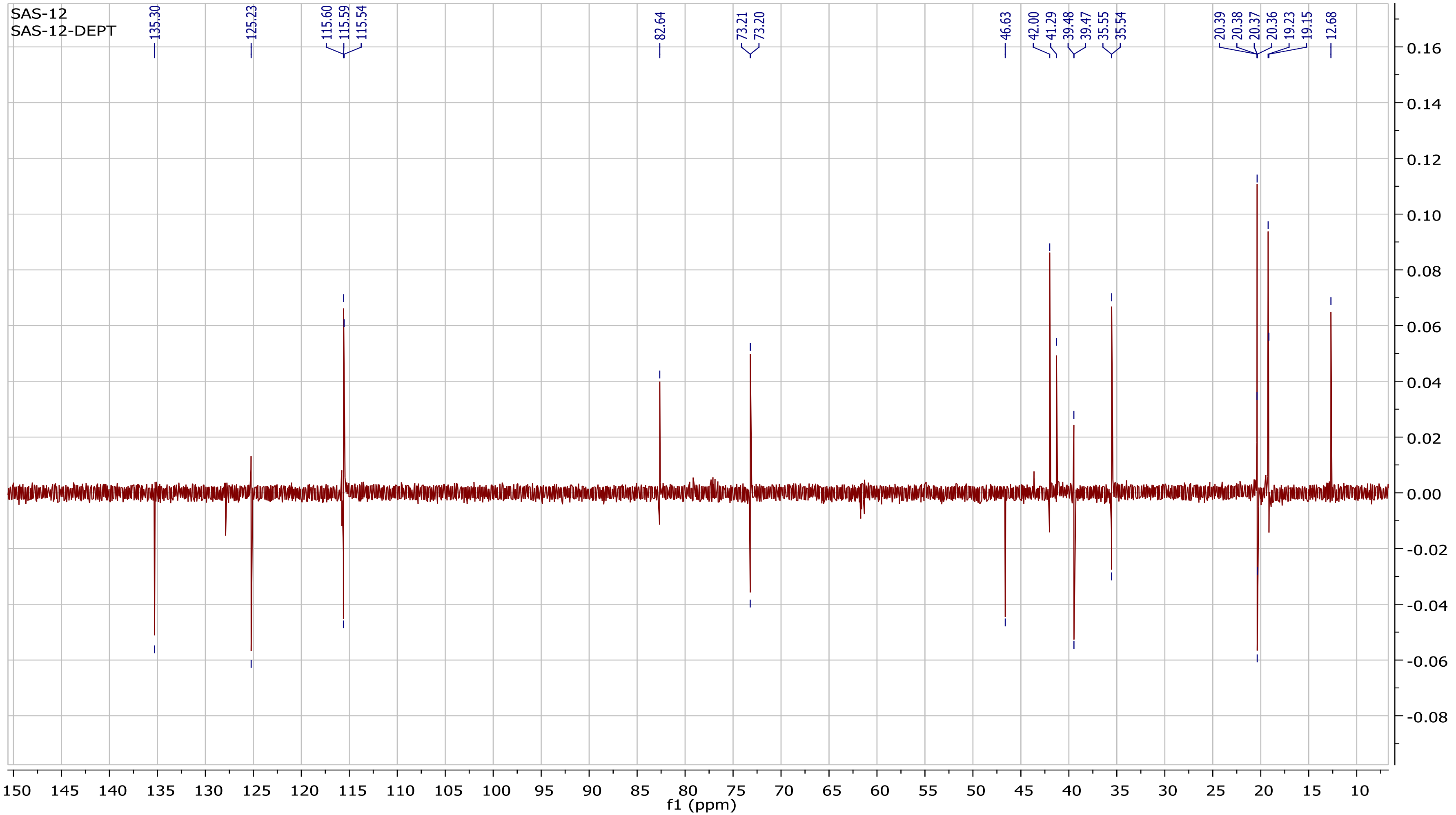

S12:DEPT-135 of **2**

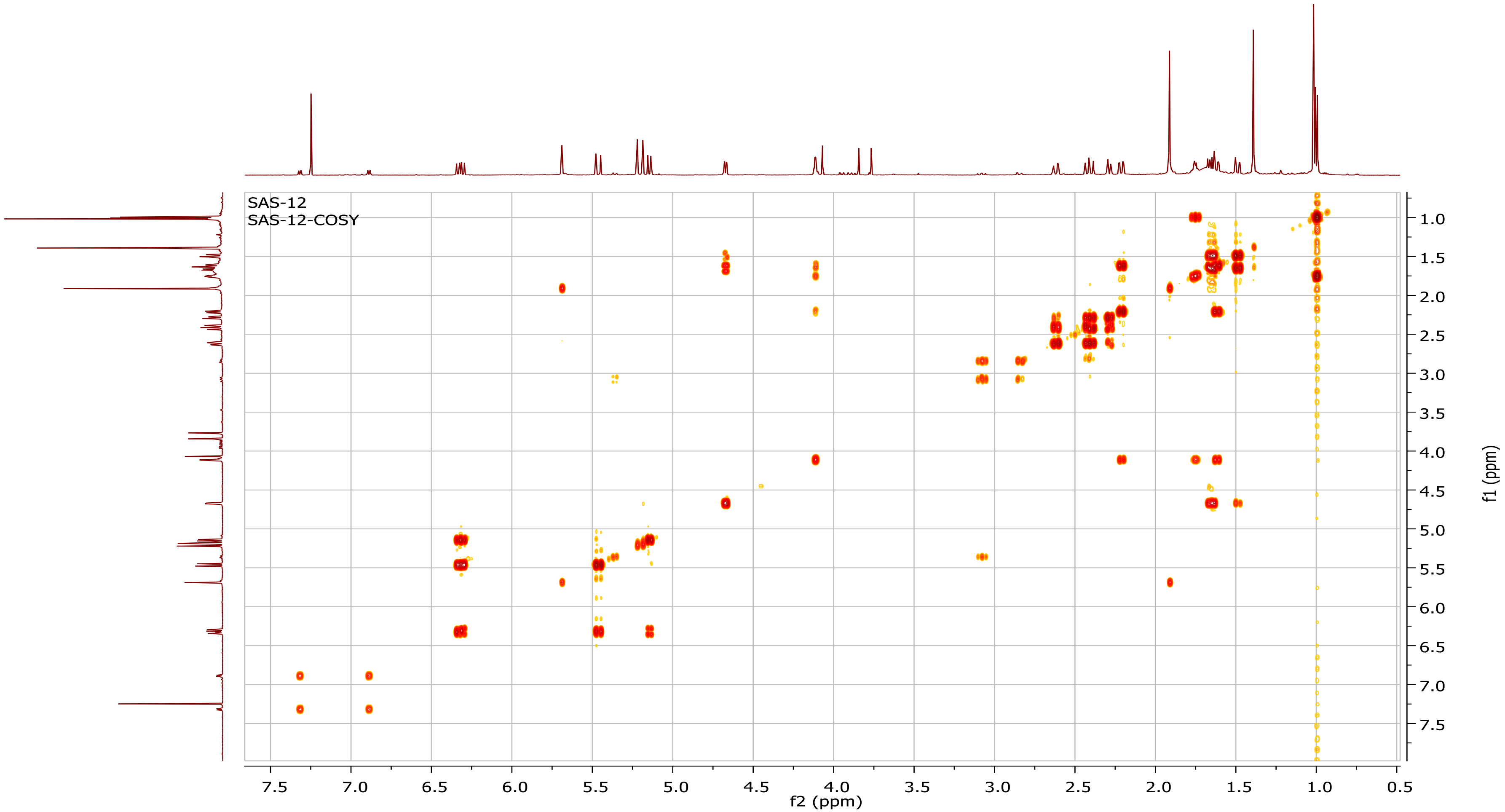

S13: ^{1}H^{1}H COSY of **2**

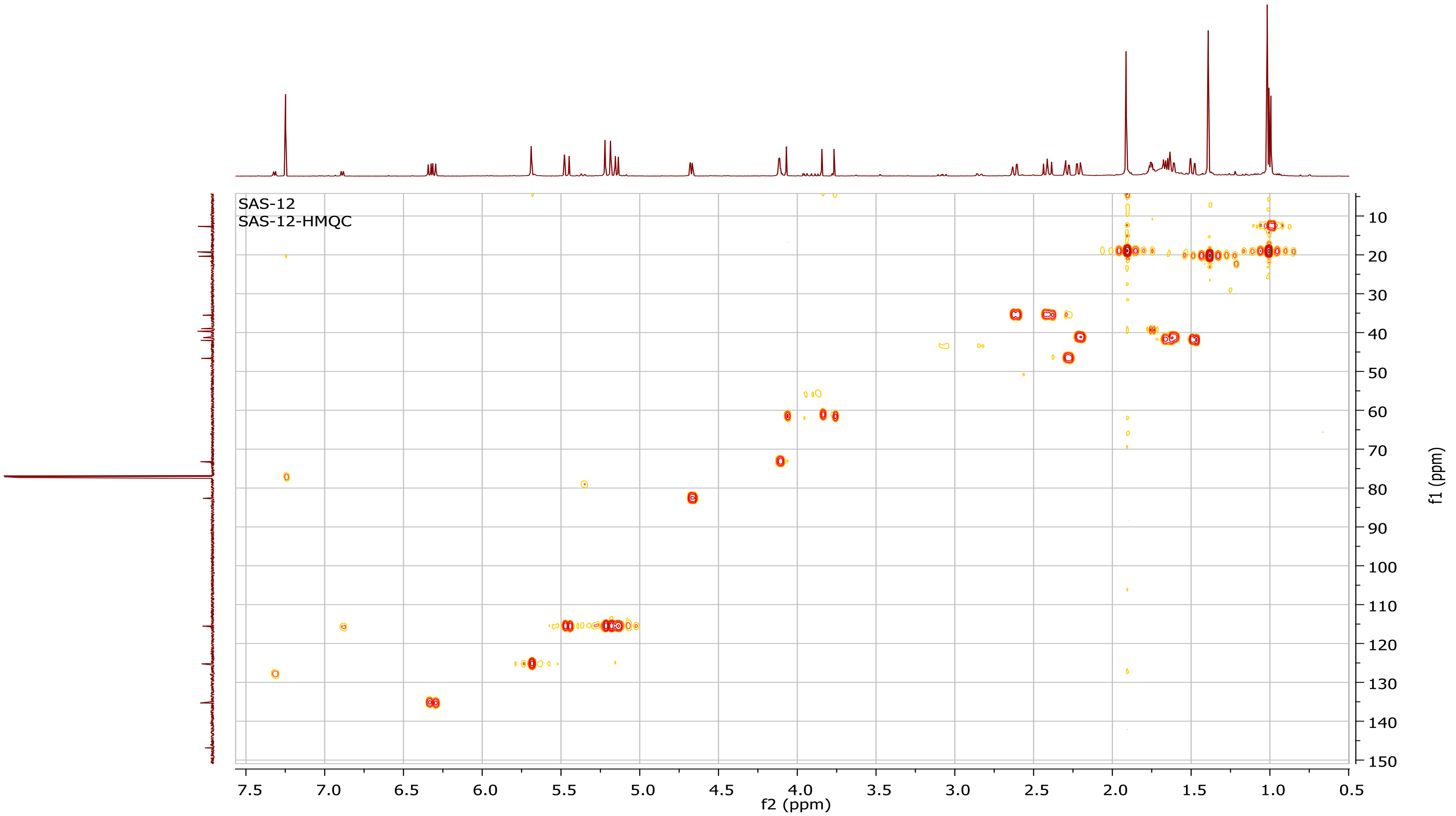

S14:HSQC of **2**

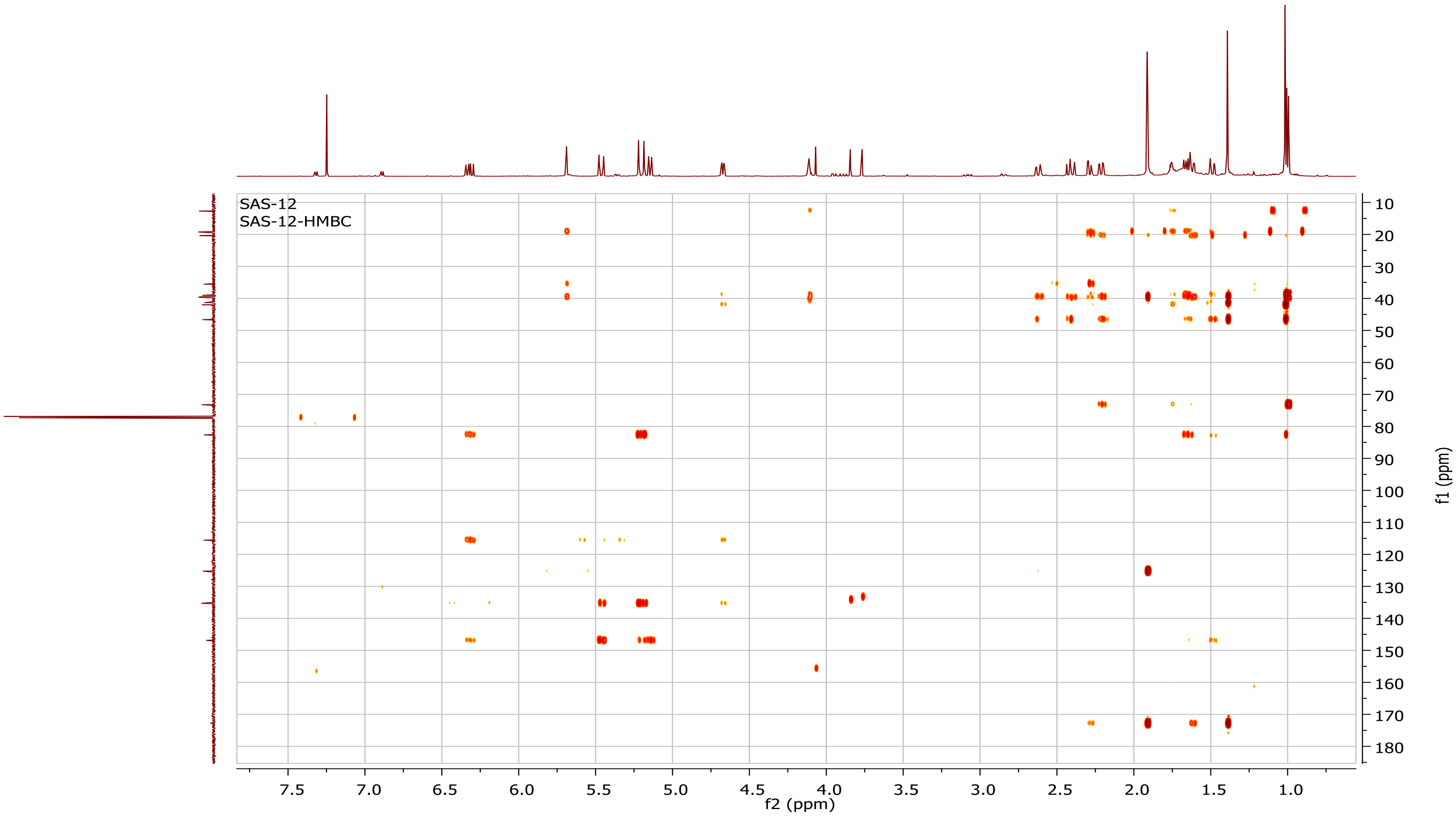

S15:HMBC of **2**

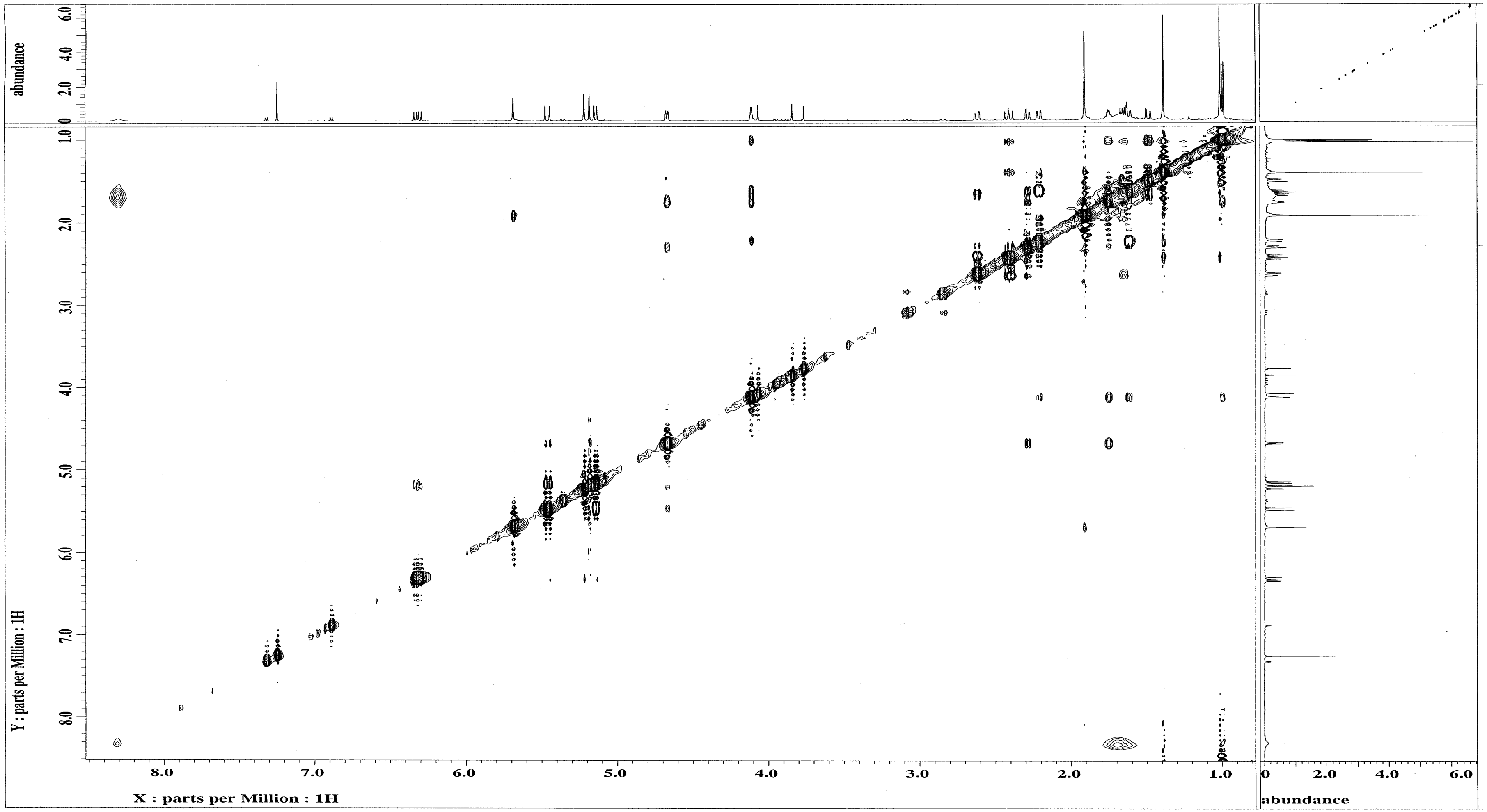

S16:NOESY of **2**

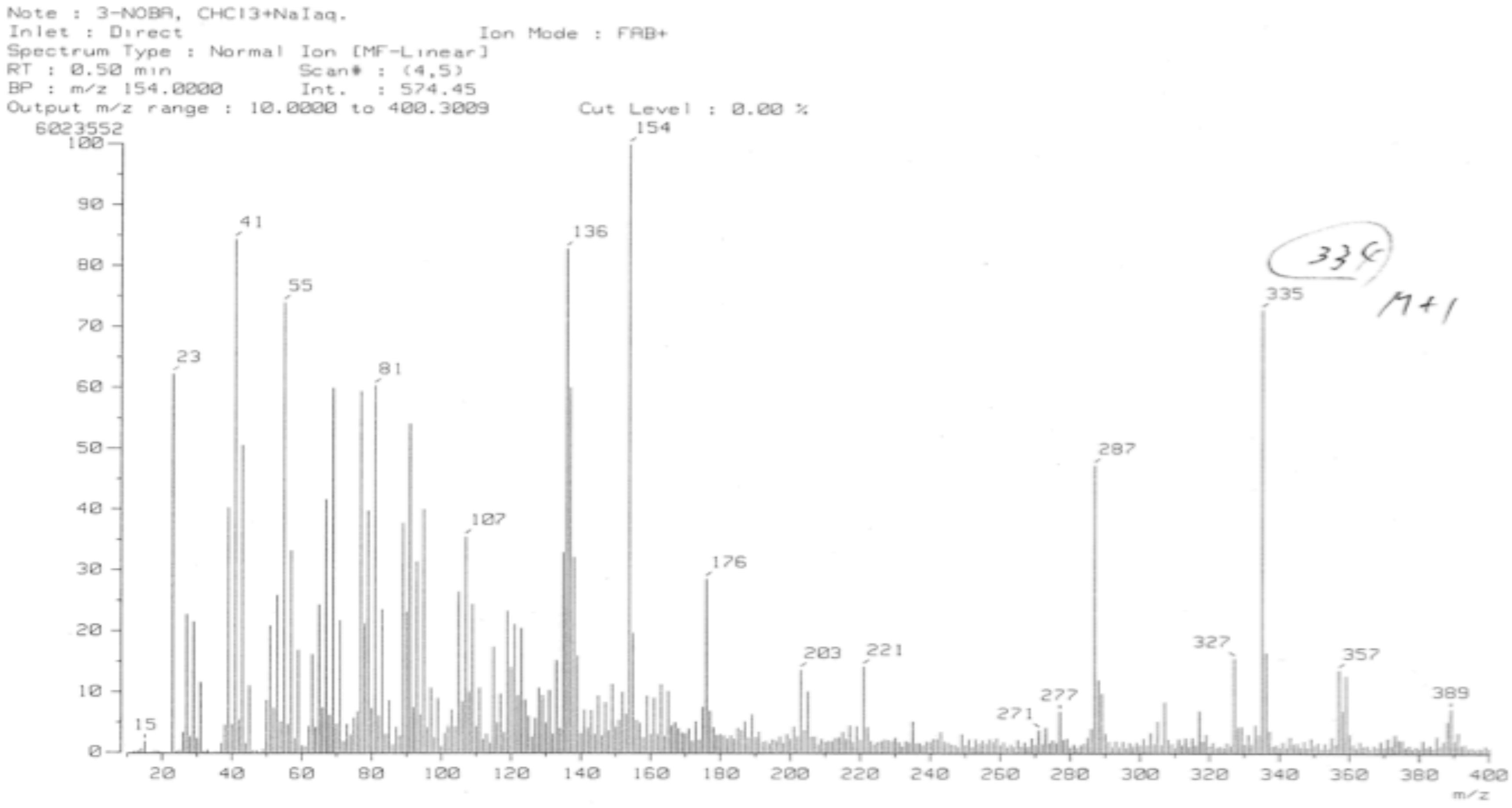

S17:FABMS of **2**

Note : 3-NOBA, CHCl3+NaIaq.
Inlet : Direct   Ion Mode : FAB+
RT : 0.90 min   Scan#: 4
Elements : C 20/0, H 40/0, O 4/0, Na 1/0
Mass Tolerance : 1000ppm, 3mmu if m/z < 3, 5mmu if m/z > 5
Unsaturation (U.S.) : -0.5 - 20.0

Observed m/z	Int%	Err[ppm / mmu]	U.S.	Composition
357.2044	16.1	+0.7 / +0.3	5.5	C 20 H 30 O 4 Na

S18:HRFABMS of **2**

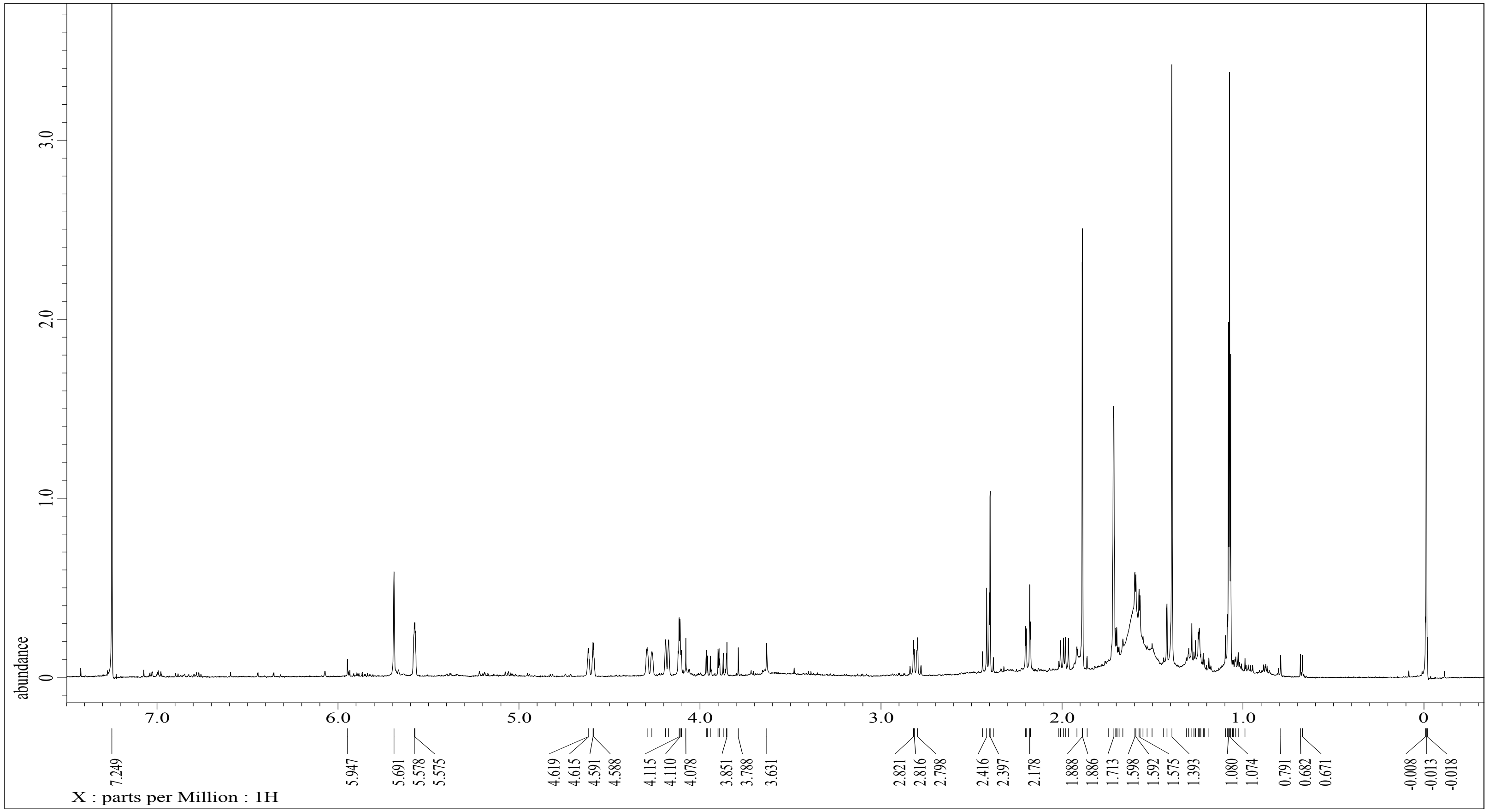

S19:^{1}H NMR of **4**

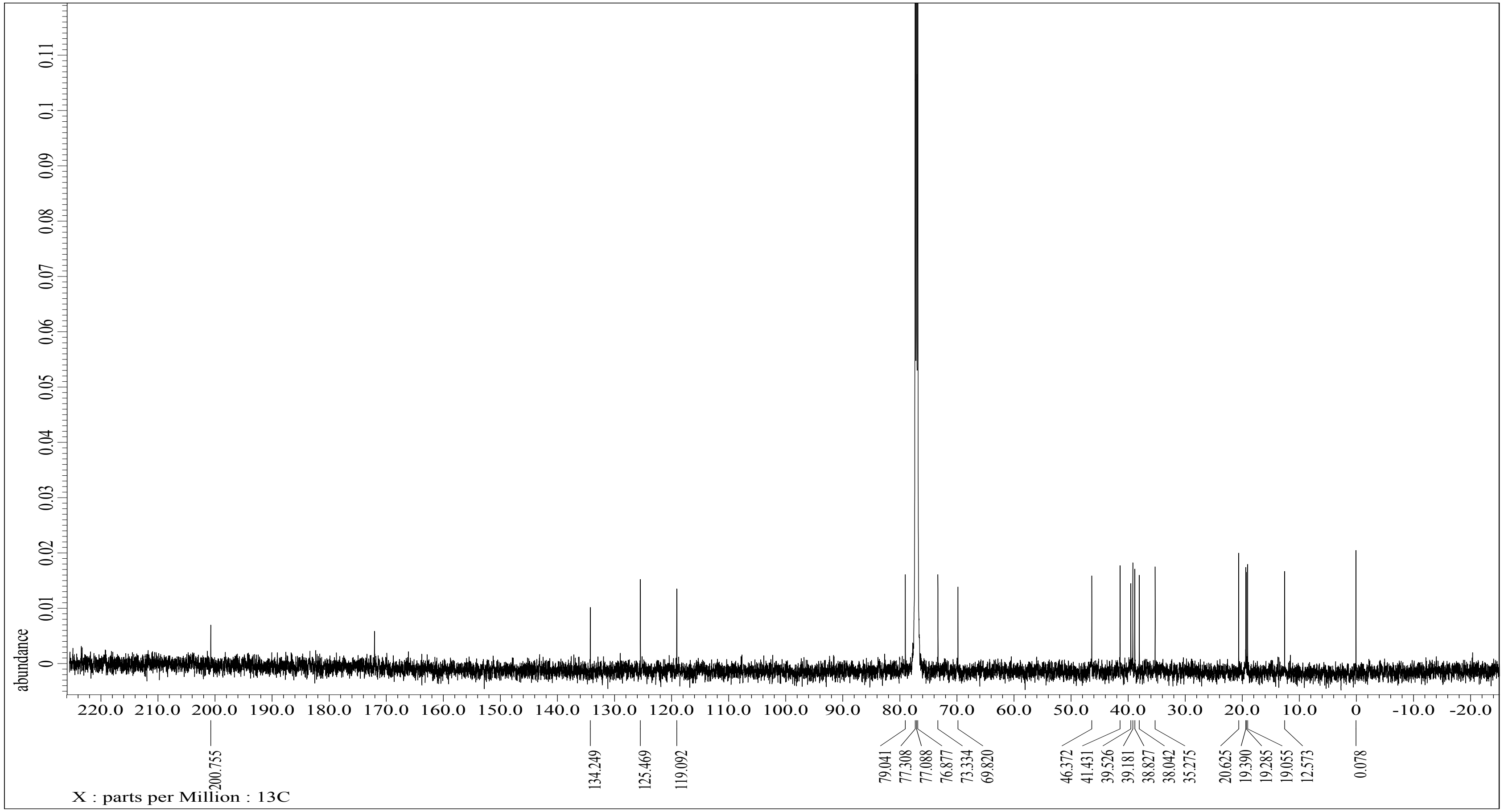

S20:^{13}C NMR of **4**

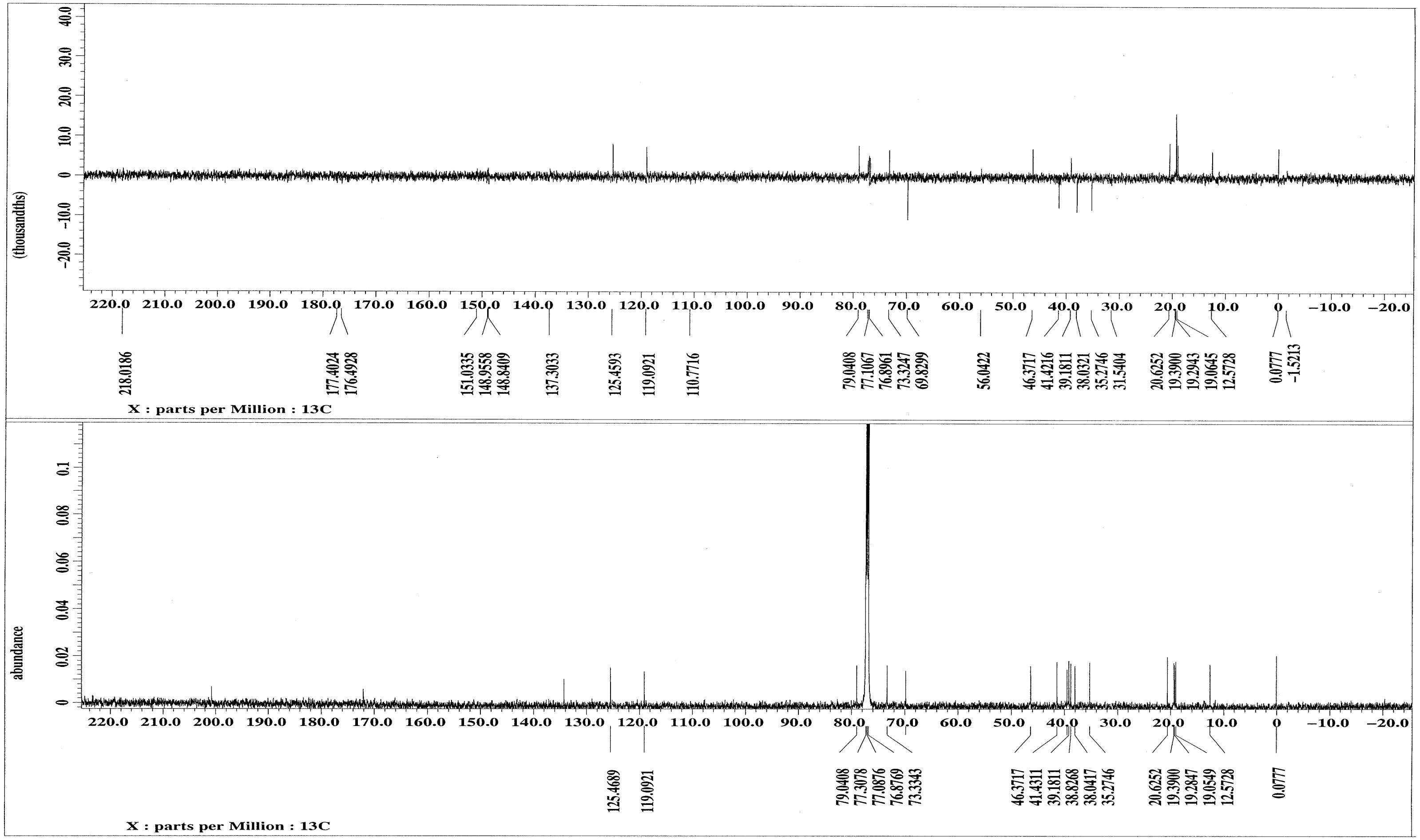

S21:DEPT-135 of **4**

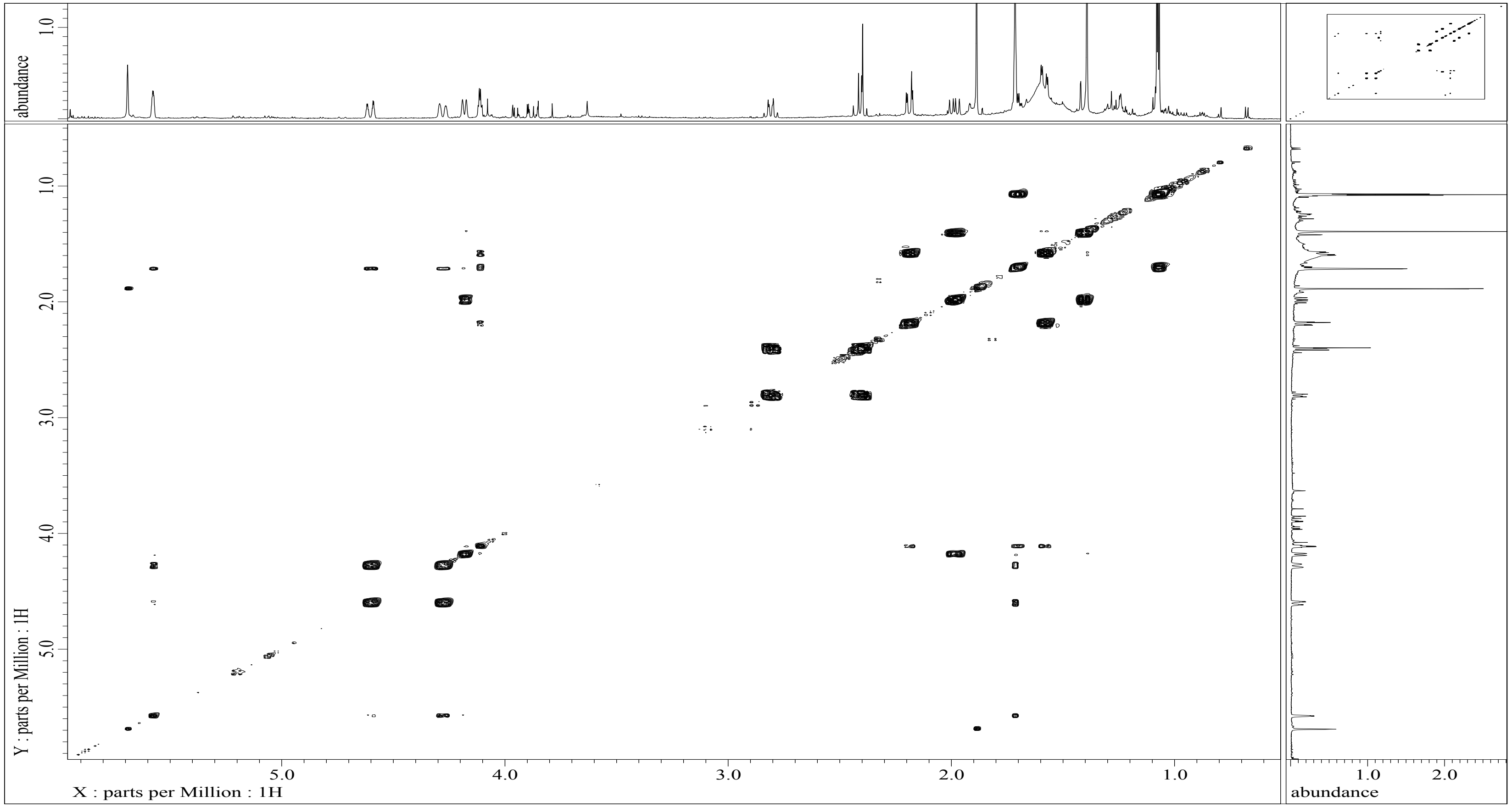

S22:^{1}H^{1}H COSY of **4**

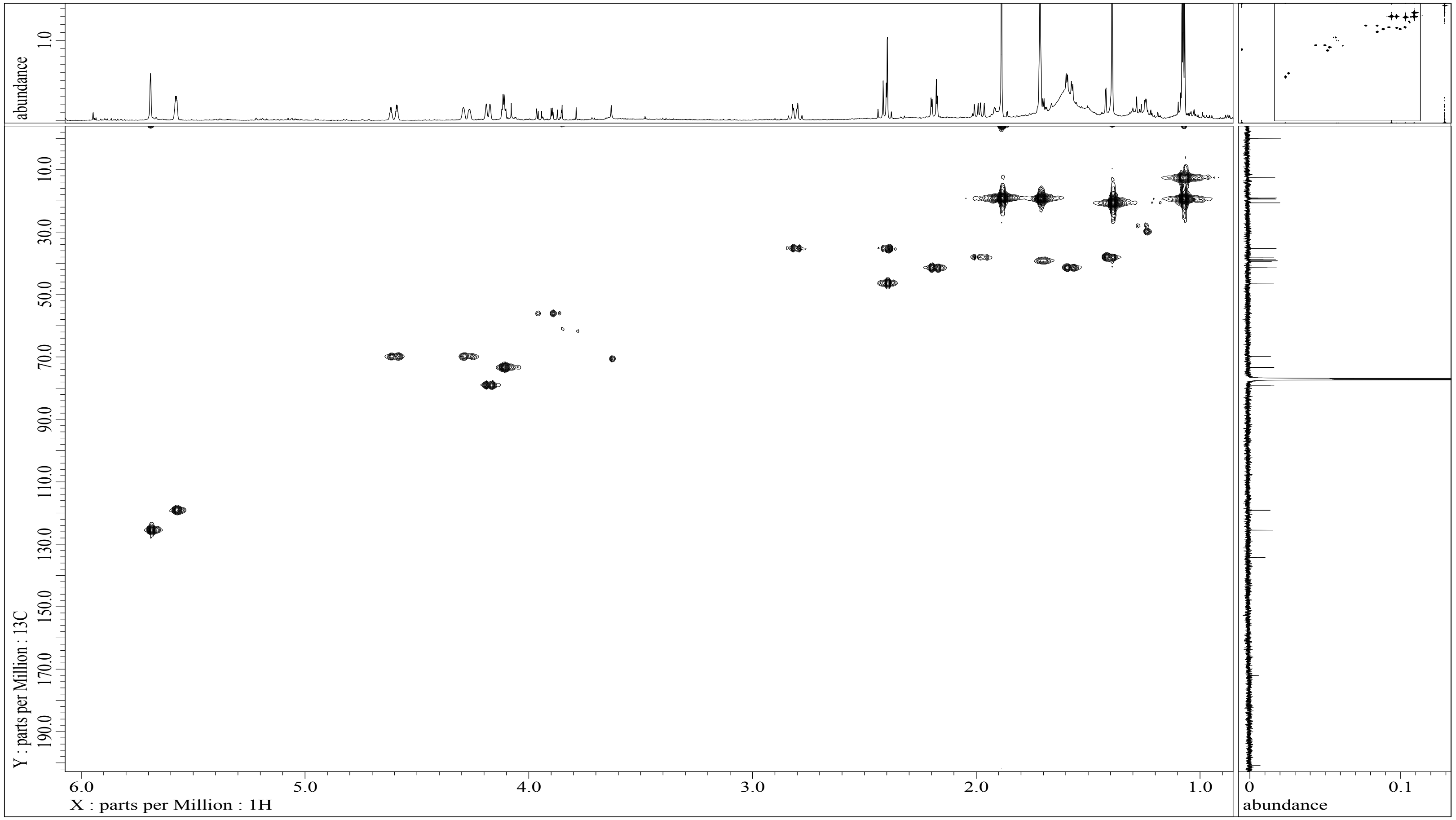
abundance
1.0
Y : parts per Million : 13C
10.0
30.0
50.0
70.0
90.0
110.0
130.0
150.0
170.0
190.0
6.0
5.0
4.0
3.0
2.0
1.0
X : parts per Million : 1H
0
0.1
abundance

S23:HSQC of **4**

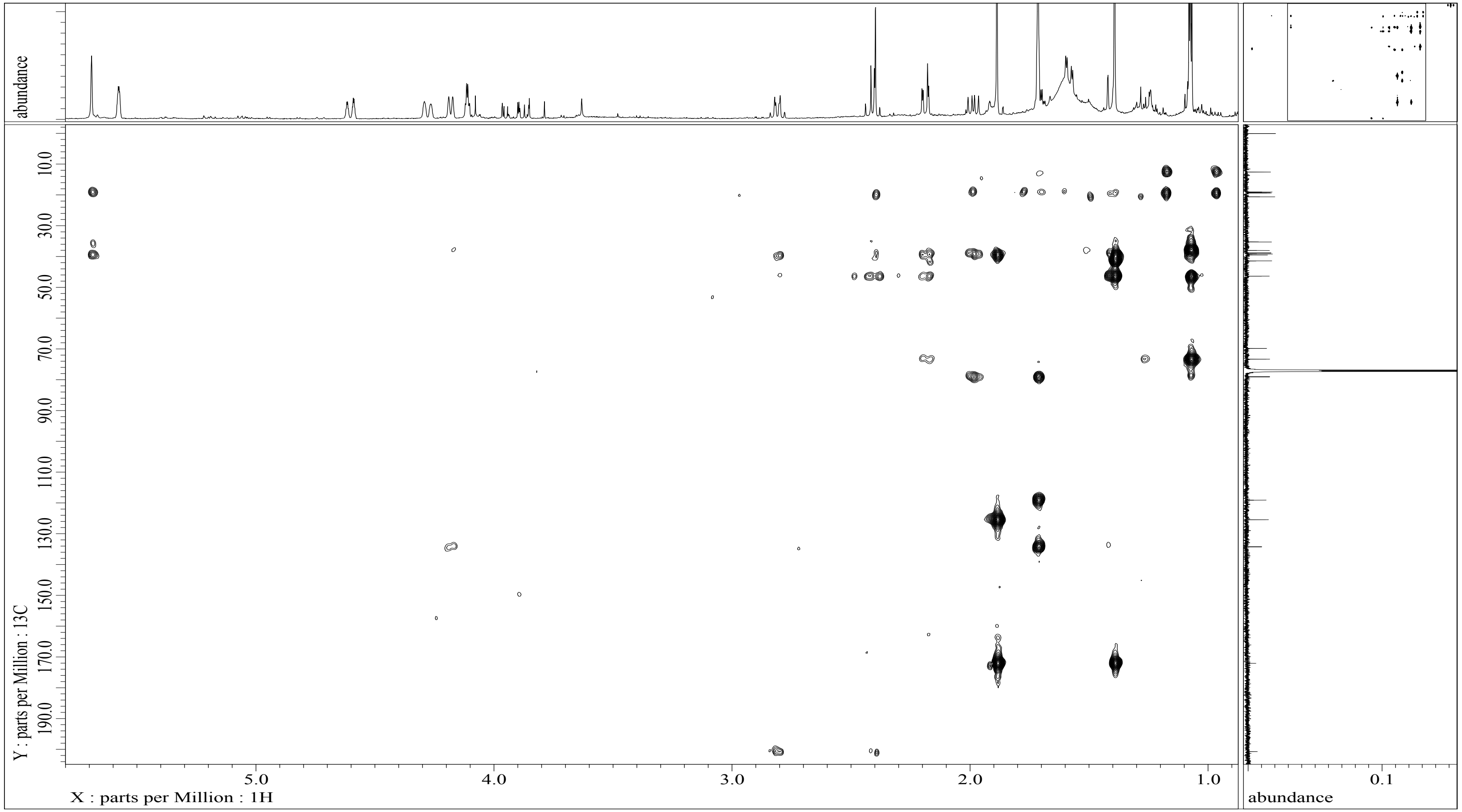

abundance
Y : parts per Million : 13C
X : parts per Million : 1H
abundance

S24:HMBC of **4**

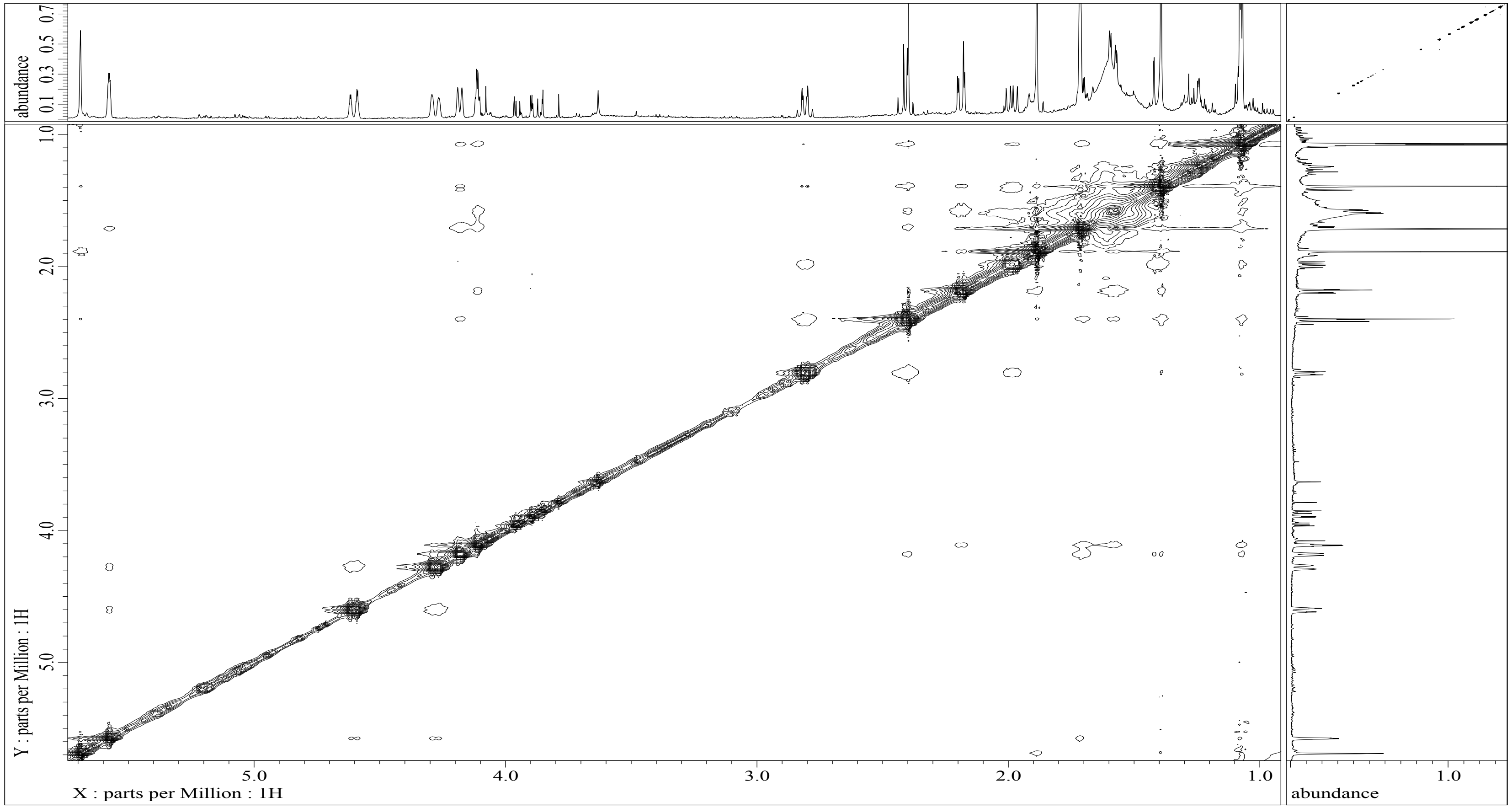

S25:NOESY of **4**

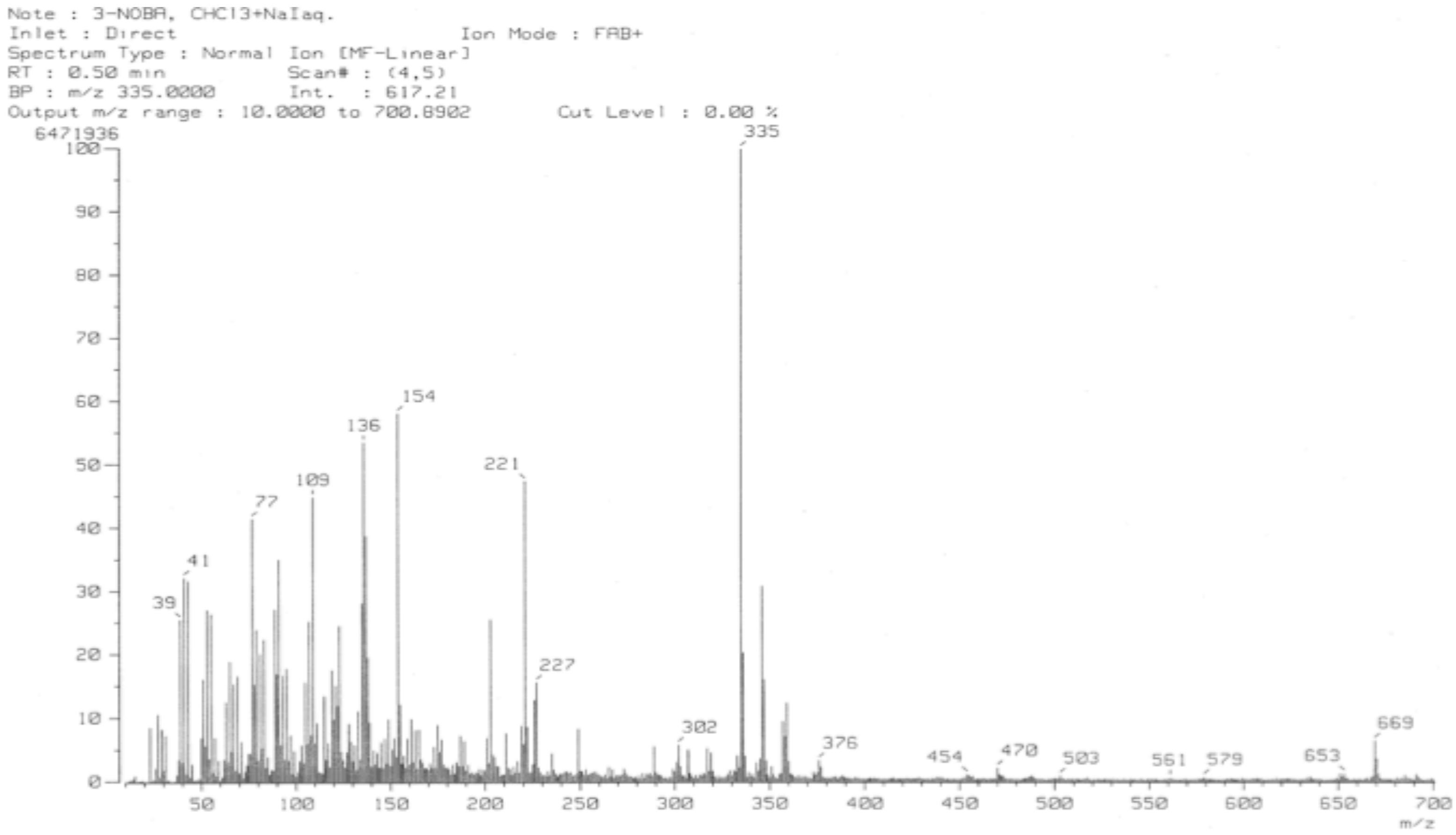

S26:FABMS of **4**

Note : 3-NOBA, CHCl3+NaIaq.
Inlet : Direct Ion Mode : FAB+
RT : 0.60 min Scan#: 3
Elements : C 20/0, H 40/0, O 4/0, Na 1/0
Mass Tolerance : 1000ppm, 3mmu if m/z < 3, 5mmu if m/z > 5
Unsaturation (U.S.) : -0.5 - 20.0

Observed m/z	Int%	Err[ppm / mmu]	U.S.	Composition
359.1143	13.9			

S27:HRFABMS of **4**
